# Zygotic vinculin is not essential for embryonic development in zebrafish

**DOI:** 10.1371/journal.pone.0182278

**Published:** 2017-08-02

**Authors:** Mitchell K. L. Han, Gerard N. M. van der Krogt, Johan de Rooij

**Affiliations:** Molecular Cancer Research, Center for Molecular Medicine, University Medical Center Utrecht, Utrecht, The Netherlands; University of Illinois at Chicago, UNITED STATES

## Abstract

The formation of multicellular tissues during development is governed by mechanical forces that drive cell shape and tissue architecture. Protein complexes at sites of adhesion to the extracellular matrix (ECM) and cell neighbors, not only transmit these mechanical forces, but also allow cells to respond to changes in force by inducing biochemical feedback pathways. Such force-induced signaling processes are termed mechanotransduction. Vinculin is a central protein in mechanotransduction that in both integrin-mediated cell-ECM and cadherin-mediated cell-cell adhesions mediates force-induced cytoskeletal remodeling and adhesion strengthening. Vinculin was found to be important for the integrity and remodeling of epithelial tissues in cell culture models and could therefore be expected to be of broad importance in epithelial morphogenesis *in vivo*. Besides a function in mouse heart development, however, the importance of vinculin in morphogenesis of other vertebrate tissues has remained unclear. To investigate this further, we knocked out vinculin functioning in zebrafish, which contain two fully functional isoforms designated as vinculin A and vinculin B that both show high sequence conservation with higher vertebrates. Using TALEN and CRISPR-Cas gene editing technology we generated vinculin-deficient zebrafish. While single vinculin A mutants are viable and able to reproduce, additional loss of zygotic vinculin B was lethal after embryonic stages. Remarkably, vinculin-deficient embryos do not show major developmental defects, apart from mild pericardial edemas. These results lead to the conclusion that vinculin is not of broad importance for the development and morphogenesis of zebrafish tissues.

## Introduction

Throughout development, cells mechanically interact with their microenvironment through sites of adhesion. These interactions govern tissue architecture and organization by coupling the contractile actomyosin cytoskeleton of cells to the extracellular matrix and to cell neighbours. These interactions are not only needed to transmit forces from outside to inside for physical deformation of cells, but these interactions are also needed for cells to sense rigidity of the extracellular environment and tension between neighbours. Tension-sensitive cell-cell junctions in the embryonic ectoderm allow, for instance, physical forces to deform cells in a coordinated manner to achieve invagination during morphogenesis [1,2]. Force-responsive cell-cell junctions also mediate cell rearrangements such as cell intercalations in *Drosophila* germband [3], and migration of an epithelial sheet over the spherical yolk cell during zebrafish epiboly [4]. Tissue tension, sensed at cell-cell contacts controls cell proliferation in *Drosophila* development [5] and force-sensitive interactions with ECM control lineage differentiation of mammalian mesenchymal stem cells [6]. Thus, the protein complexes that form adhesion sites are not only crucial for physical contact formation and maintenance, but also for sensing forces and eliciting appropriate intracellular biochemical responses during the dynamic development of a multicellular organism or tissue. This latter function is called mechanotransduction.

Vinculin is a key player in mechanotransduction at adhesion complexes. It is an integral member of integrin adhesions, which couple the actomyosin cytoskeleton to the ECM [7]. In these adhesions, a key interactor of vinculin is talin, which forms the link between integrins and actomyosin. In addition, the vinculin-binding sites in talin are regulated by tension [8]. Specifically, the affinity increases > 100 fold when tension rises above 5pN per talin molecule [9]. The result of this tension-sensitive recruitment of vinculin to talin at integrin-based adhesions is a reinforcement of the interaction of the adhesion complex with actomyosin, and a remodelling of the integrin-associated actomyosin [10,11]. Similarly, vinculin is recruited to cadherin-based cell-cell junctions with increasing tension, thereby strengthening the adhesion [12,13]. Vinculin binds the adherens junction component α-catenin, which interacts with cadherin-bound β-catenin through its N-terminal head domain, and links the cadherin complex to F-actin using its C-terminal tail. Tension in excess of 5 pN induces unfolding of α-catenin, which leads to the exposure of its vinculin-binding domain [14]. Recruitment of vinculin then locks α-catenin in the unfolded state and promotes adhesion strengthening through binding of other actin-binding proteins such as ZO-1, afadin and vinculin itself [15]. Attenuating vinculin recruitment by mutation of its binding site in α-catenin results in reduced cell-cell contact integrity during junction remodelling in endothelial cell [16], as well as a reduced rate of epithelial barrier formation [17]. In addition, loss of the mechanosensitive vinculin-binding domain of α-catenin induces strong convergent extension defects during gastrulation [18]. Notably, besides α-catenin- and talin-dependent tension-induced recruitment, vinculin also binds to other proteins at both integrin and cadherin adhesions such as paxillin and β-catenin [19]. These interactions also contribute to adhesion stability [20–23].

Despite the well described function in adhesion mechanotransduction in cell culture models, the role and importance of vinculin *in vivo* remains controversial. Vinculin-deficient *C*. *elegans* show defects in embryonic elongation and die in larval stages due to defective muscle function [24]. In *Drosophila*, vinculin mutants are perfectly viable and show very mild muscle defects [25,26], while expressing hyper-active vinculin isoforms in this vinculin-null background does induce morphogenetic defects and lethality [27]. In mice, loss of vinculin induces defects apparent from day E8, and is embryonic lethal by day E10 [28]. Problems in heart development were found–hearts were smaller and contained less myocytes—and embryos were small, showed retarded growth of limbs and somites, and failed to close the neural tube. This indicates that vinculin plays a role in the cell rearrangement movements during morphogenesis in multiple tissues. In mice where one allele of vinculin was disrupted, embryogenesis occurred without gross defects, but a large percentage of mice developed stress induced cardiomyopathies [29]. A protective role for vinculin in heart function was further established using cardiomyocyte-specific vinculin depletion. These mice died from sudden heart failure caused by ventricular arrhythmias or developed dilated cardiomyopathy [30]. Conversely, upregulation of vinculin and associated proteins was found during aging in hearts of monkeys and flies, and this was shown to correlate with increased lifespan [31]. Indeed, vinculin is expressed in skeletal and cardiac muscle and localizes to the intercalated discs and costameres [32–34]. There are a few reports on VCL mutations in individuals with dilated and hypertrophic cardiomyopathy, but these remained incidental and no solid causal relationship could be established (see NCBI/OMIM database for details). It became clear from these studies that vinculin is important for the correct development and maintenance of muscular junctions, but how this relates to its function as a mechanotransducers at sites of adhesion is unknown. It should be noted that a splice variant of vinculin, named metavinculin is specifically expressed in muscle cells, including cardiomyocytes, and that it might be a specific function of metavinculin that is needed in muscular junctions [35]. Because of the specific role of vinculin in mechanotransduction at cell-ECM and cell-cell junctions, it is anticipated that depletion of vinculin would broadly affect development of epithelial tissues, where forces applied on cell-cell junctions mediate key cell migrations in development, such as convergence extension movements [3], primordial germ cell positioning [36] and development of the vascular network [37].

To investigate the importance of vinculin during morphogenesis we used the zebrafish as a model system, as the development and morphogenesis of many organs and tissues, including the skin [38], vasculature [37], and heart, [39] can be easily tracked by microscopy. In this study, we show that the zebrafish genome contains two vinculin isoforms, vinculin A and vinculin B, which both show very high sequence conservation with higher vertebrate orthologues in key regions of vinculin functioning. Using TALEN and CRISPR-Cas gene editing technology we generated vinculin-deficient zebrafish. Single vinculin A mutants do not show any major developmental defects and are fully viable and reproductive. Surprisingly, zygotic vinculin A/vinculin B double mutants also do not show major defects during embryonic development. While these mutants develop mild pericardial edemas, skeletal muscles appear normal. Furthermore, they are fully viable during the first 5 days of development when most tissue and organs have been formed and are functional. Nevertheless, fish depleted from zygotic vinculin do not survive until adulthood, which is likely due to the loss of *vclb*, which was recently found to cause lethality in juvenile stages associated with defects in the coronary vasculature [40]. We conclude that vinculin is mostly dispensable for embryonic development in zebrafish, but crucial for adult life.

## Materials and methods

### Fish lines and husbandry

Zebrafish were housed at the Hubrecht Institute, Utrecht, the Netherlands according to local guidelines and policies in compliance with national and European law. The following fish lines were used: Tupfel Longfin (TL) wild-type and vinculin gene-depleted mutants *vcla*^*hu10818*^ and *vcl*^*hu11202*^. From the mutant parental lines the following lines were established: Zygotic *vcla*^*-/-*^, Maternal Zygotic *vcla*^*-/-*^, Zygotic *vclb*^*+/-*^ and Maternal Zygotic *vcla*^*-/-*^*vclb*^*+/-*^. Fish were maintained according to standard laboratory conditions. Animal experiments were approved by the Animal Experimentation Committee (DEC) of the Royal Netherlands Academy of Arts and Sciences.

### Cell lines and cell culture

Madin Darby Canine Kidney cells (MDCK strain II) epithelial cells depleted from α-catenin, and HEK293T cells were cultured in high glucose DMEM containing L-glutamine, supplemented with 10% Fetal Calf Serum and Penicllin/Streptomycin in standard 10 cm culture dishes. Cells were transfected (with murine α-catenin-GFP or α-cateninΔVBS-GFP in the MDCKs in combination) with either zebrafish vinculin A-GFP or vinculin B-GFP, using X-tremegene9 (Roche) as per manufacturer’s instructions.

### Antibodies

For Western blotting, the following antibodies were used: mouse anti-vinculin (Sigma; hVin1; 1:2000), anti-β-actin (Sigma; AC74; 1:5000) and rabbit anti-GFP (Sigma; 1:2000).

### Characterization of the *vclb* locus

The *vclb* locus was found using a translated nucleotide blast with mouse vinculin (NCBI Ref. Seq. NM_009502) as a protein query. The *vclb* locus was not annotated formally in the Zv9 zebrafish genome database, but did contain a GENSCAN prediction sequence. This was used to generate primers to clone the gene from cDNA, generated from total mRNA of whole embryo lysates. Forward: 5’- ATGCCGGTTTTCCACACGAAGAC-3’ and reverse: 5’-TCACTGGTACCAGGGTGTCTTGC-3’. The resulting PCR product was cloned into pGEM-T using TA-cloning and fully sequenced.

### Alignments of vcl homologs

The vinculin sequences of various animals were aligned and compared using Clustal Omega in the Jalview web app software (Waterhouse AM 2009). Used NCBI reference sequences: *Drosophila* (NP_476820.1), *C*. *elegans* (NP_501104.2), Human (NP_003364.1), Mouse (NP_033528.3), Chicken (NP_990772.1), *Xenopus* (NP_001090722.1).

### Generation of *vcla* TALEN constructs

TALENs targeting the *vcla* locus were designed using the TALE-NT tool (https://tale-nt.cac.cornell.edu/node/add/talen) using the guidelines specified in [41]. The chosen optimal target sequence in exon 4:

5’-TTGTGGAAACCATGGAGGACTTGATCACTTACACTAAAAACCTGGGACCAGGTA-3’ (binding sites of the Left and Right TALEN arms are underlined) contains a BclI (Promega) restriction enzyme site (TGATCA). TALENs were generated using the Golden Gate kit in combination with the obligate heterodimeric FokI pCS2TAL3DD (Addgene, #37275) and pCS2TAL3RR (Addgene, #37276) backbones [42].

TALEN targeting plasmids were linearized with NotI (Promega) after which IVT mRNA was generated using the mMessage mMachine SP6 kit (Ambion), and purified using the RNeasy mini kit (Qiagen).

### Generation of *vclb CRISPR-Cas constructs*

CRISPR guide RNA oligonucleotides were designed by targeting exon 1 of *vclb* using the ZiFit design website (http://zifit.partners.org/ZiFiT/). Oligonucleotides 5’- TAGGCCCAGCAGATCTCCCATC-3’ and 5’- AAACGATGGGAGATCTGCTGGG-3’ were annealed and cloned into pDR274 (Addgene, #42250). The construct was linearized using DraI (NEB) and guide RNA was transcribed using the T7 MEGAshortscript kit (Ambion). Cas9 mRNA was generated from NotI linearized pCS2-nCas9n (Addgene, #47929) using the mMessage mMachine SP6 kit (Ambion), and purified using the RNeasy mini kit (Qiagen).

### Microinjection of constructs

Left and Right *vcla* TALEN arm mRNA (10, 25 and 50 pg each) or 15 pg *vclb* guide RNA together with 150 pg nCas9n mRNA were diluted in nuclease-free water containing phenol red, and injected into the blastomeres of one-cell stage zebrafish embryos in a volume of 1 nl.

### Genotyping of mutant alleles

To isolate genomic DNA, zebrafish embryos or fin clips were incubated in 50 μl or 100 μl embryo lysis buffer respectively, for 60 minutes at 60°C. Proteinase K was heat-inactivated by incubating the samples for 15 min at 95°C. The genomic DNA was used as template to amplify the *vcla* TALEN and *vclb* CRISPR-Cas target loci. Primers flanking the target site of *vcla*: VclA E4 forward, 5’- TCTGCATTGACGTATCATCGAC-3’ and VclA E4 reverse, 5’- ACTCGTGCAAACAGTTGCTG-3’. Primers flanking the *vclb* target site: VclB 5UTR forward, 5’- CTCTCTGTGTAGGAGCTCTGC-3’ and VclB E1 reverse, 5’- GGATGGCTTTCCCGTCC-3’.

To assess *vcla* mutant alleles, the PCR amplicons were incubated with BclI (Promega) at 50°C for 2 hrs, resulting in the generation of 504 bp and 179 bp fragments. Successful mutagenesis was detected by loss of the restriction recognition site and the presence of an undigested band of 683 bp. Mutant alleles were sequenced using primer VclA E4 forward, 5’- TCTGCATTGACGTATCATCGAC-3’.

CRISPR-Cas mediated *vclb* mutant alleles were detected by sequencing the PCR amplicon of the target site using primer VclB 5UTR forward, 5’- CTCTCTGTGTAGGAGCTCTGC-3’.

### Generation of cDNA using RT-PCR

RNA was isolated from 20–30 embryos using Trizol (Invitrogen). The purified RNA was used to generate cDNA using the SuperScript III First Strand Synthesis kit.

### Immunohistochemistry and cytoskeletal washout

For immunocytochemistry in cells, MDCK cells were washed 1 time in PBS, 1 time in CSK buffer (300 mM Sucrose, 0.5% TX-100, 10 mM Pipes pH 7, 50 mM NaCl, 3mM CaCl2, 2 mM MgCl2) and 1 time in PBS before fixation using 4% paraformaldehyde in PBS for 20 min. After fixation, cells were permeabilized with 0,4% Triton X-100 in PBS for 5 minutes and blocked in 2% BSA for 1 hour. Phalloidin-415 (Promokine was diluted in 2% BSA and incubated with the cells for 1 hour. Afterwards, cells were mounted in Mowiol 4–88/DABCO solution (Sigma-Aldrich).

For the staining of zebrafish muscles, 5 dpf embryos were fixed in 2% PFA in PBS overnight. Embryos were then washed 3–5 times 5 minutes in PBT (PBS + 0.1% Triton-X100). Embryos were blocked in PBT +10% Normal Goat Serum for 1 hour, then incubated O/N with Phalloidin-Alexa594 (Life Technologies). Samples were then washed 4x 30 min in PBT. The head was cut off for genotyping, while the posterior half of the body was mounted on cover slips in mowiol.

### Microscopy and live imaging

Live embryos at were imaged using a Leica MZFLIII upright microscope. Fixed immunostained samples were imaged on a Zeiss LSM880 confocal microscope fitted with a 20x/0.75 NA objective.

### Stastistics

Statistics was performed using GraphPad Prism software. Differences between two groups were assessed using a two-tailed paired t-test.

## Results

### The zebrafish genome contains two vinculin isoforms

**Fig 1:**
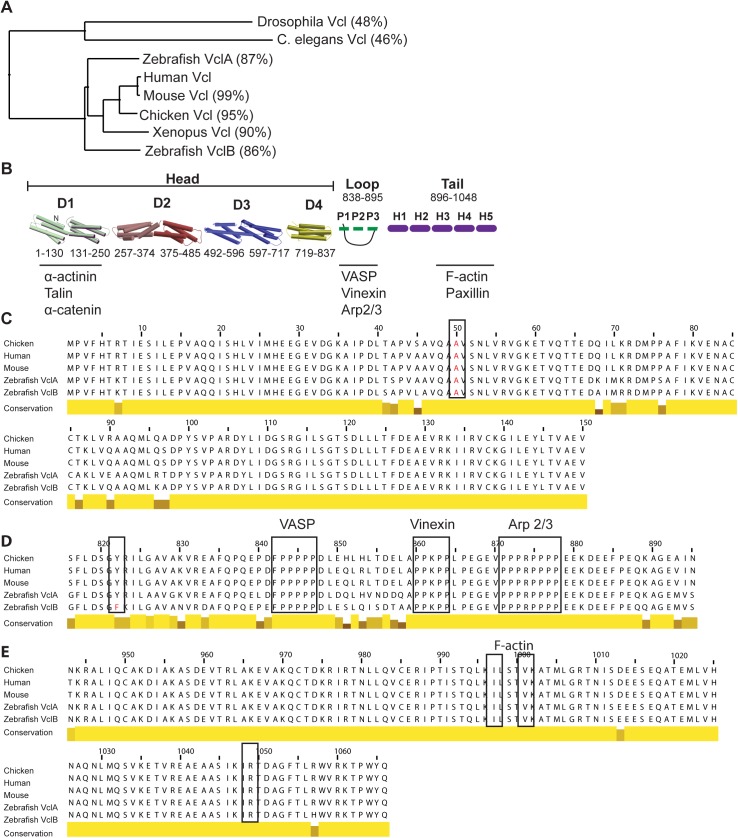
Comparison of zebrafish vinculin isoforms. (A) Phylogenetic analysis of the zebrafish vinculin genes with vinculin from other model systems based on the percentage identity on amino acid level. Percentage identity (in parenthesis) shown is compared to the human sequence. Used NCBI reference sequences: *Drosophila* (NP_476820.1), *C*. *elegans* (NP_501104.2), Human (NP_003364.1), Mouse (NP_033528.3), Chicken (NP_990772.1), *Xenopus* (NP_001090722.1). The protein sequences of the zebrafish vinculin isoforms were based on our own cDNA clones. (B) Schematic representation of vinculin protein structure. Binding partners of key regions are shown. (C-E) Multiple sequence alignment of vertebrate vinculin isoforms using ClustalOmega. Amino acid conservation is shown as bars underneath the alignment, with higher conservation shown as higher yellow bars, and lower conservation as lower darker yellow bars. Key regions are highlighted using bounded boxes. The alignment in (C) shows the first four helices of the D1 head domain, the alignment in (D) shows the loop domain, and the alignment in (E) shows the last three helices of the tail region.

Zebrafish are members of the teleost family, which have undergone an additional genome duplication event compared to other vertebrates [43]. A translated nucleotide BLAST using the mouse vinculin protein sequence (NCBI Ref. Seq. NM_009502) revealed two vinculin orthologues present in the zebrafish genome. The first isoform, *vinculin a (vcla)* present on chromosome 13, was well annotated, while its paralog *vinculin b* (*vclb*) on chromosome 12 was not (as of assembly Zv9). Using a GENSCAN prediction sequence the cDNA for *vclb* was cloned and compared to known vinculin protein sequences to analyze conservation (S1 Table and S1 Text). Both zebrafish *vcla* and *vclb* show a high sequence conservation at the protein level (87% and 86% identical amino acids respectively) with mammalian *vcl* (Fig 1A).

We further examined the three key regions (Fig 1B), containing the sites of interaction or regulation involved in vinculin functioning, using multiple sequence alignment (Clustal Omega) with the most studied vinculin homologs from higher vertebrates. The first four helices of the vinculin head D1 domain are highly conserved across vertebrates (Fig 1B and 1C). This domain has been shown to be essential in binding talin [44], α-catenin [45] and α-actinin [46]. The key residue of which mutation perturbs all of these interactions, A50, is fully conserved [47,48]. This suggests that both vinculin A and vinculin B can interact with the same partners as in higher vertebrates.

Zebrafish vinculin A contains one extra lysine at position 262 (not shown), but the numbering for the following alignments skips this residue for the sake of uniformity among amino acid positions between the different species. The loop regions in vinculin A and B are a little less conserved across the higher vertebrate homologs, but the proline rich sequences, containing binding sites for VASP [49], vinexin [50], and Arp2/3 [51] are fully conserved, indicating that these interactions are conserved as well (Fig 1B and 1D). Finally, the Vinculin tail is also highly conserved. Especially Helices 3–5 which mediate paxillin and F-actin binding (Fig 1E) [52,53] are almost completely identical. A larger splice isoform normally expressed in muscle tissue called metavinculin, contains an extra exon (exon 19), coding a 68-residue insert between Helices 1 and 2 of the Vinculin tail, which alters its F-actin dynamics [54]. This extra exon is indeed annotated for vinculin A in the Zv9 genome database, but full annotation of the vinculin B genomic region spanning this exon was missing. We did not detect the extra exon in the cDNA of vinculin A or B extracted from whole 1dpf embryo lysates, corroborating a recent article on vinculin B function in zebrafish [40].

Recently described phosphorylation sites involved in vinculin functioning are also conserved across both isoforms, such as Y100, S1033, S1045 and Y1065 [55–57]. The one notable difference between zebrafish vinculin A and B is the change of the otherwise conserved Y at position 822 to F in vinculin B. The phosphorylation of this tyrosine was found to be crucial for the interaction of vinculin to β-catenin and localization to cell-cell junctions in MCF10A cells, although it is localized outside of the β-catenin interacting domain [58]. The replacement by a phenylalanine completely abrogates the possibility of phosphorylation or other means of inducing hydrogen-bonding at this position. In fact, the mutation of this tyrosine to phenylalanine in chicken vinculin abrogates β-catenin interaction, and cell-cell junction localization, leading to a reduced stability of cadherin-based cell-cell junctions in knock-down rescue experiments. This would suggest that vinculin B is a vinculin isoform with impaired functionality in cell-cell junction stabilization. Thus, the most critical regions for vinculin’s main interactions are conserved in zebrafish vinculin A and B, but vinculin B lacks a crucial regulatory tyrosine involved in cell-cell adhesion.

**Fig 2:**
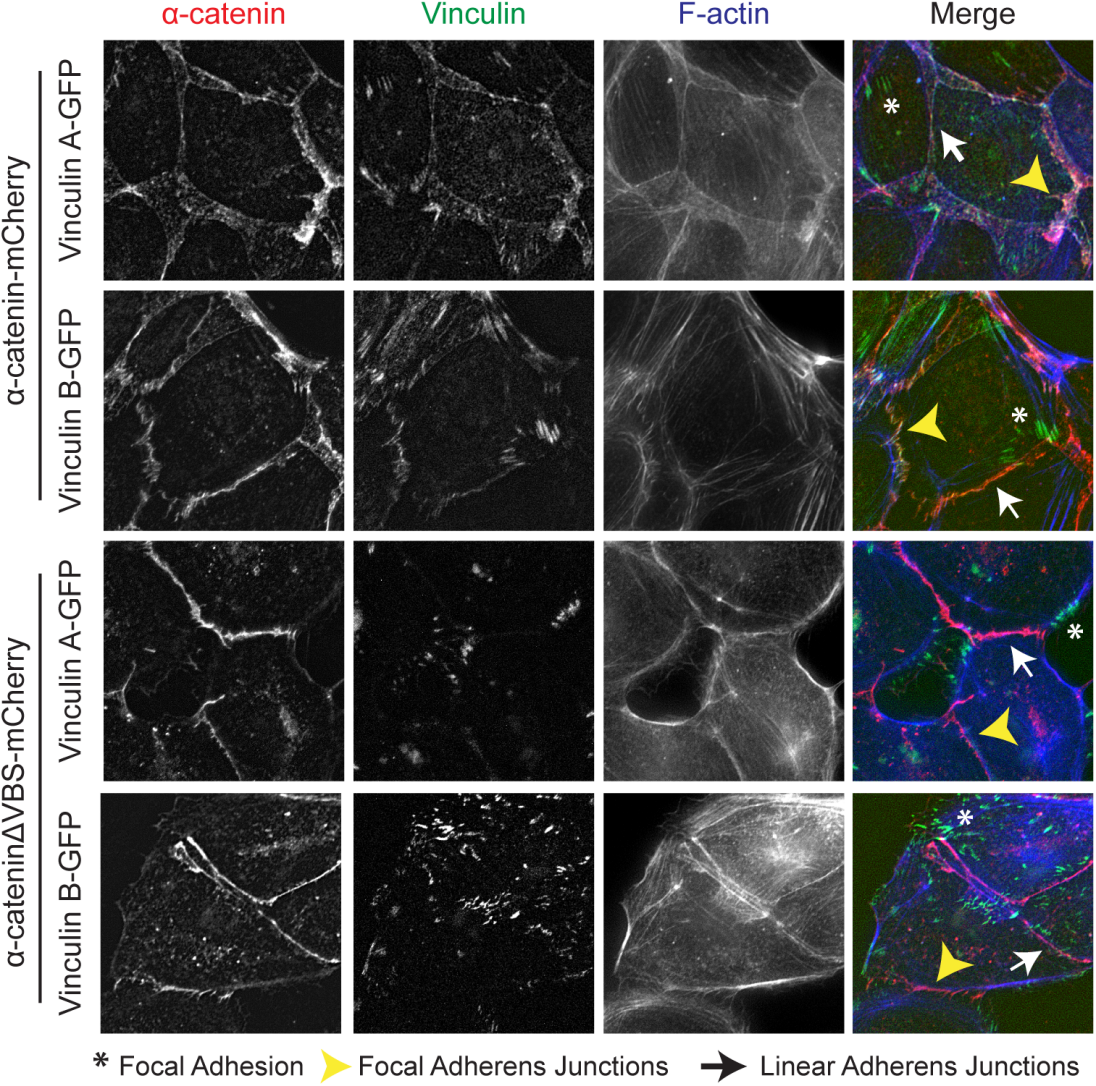
Zebrafish vinculin A and vinculin B localization. Fixed α-catenin-depleted MDCK epithelial cells expressing either α-catenin-mCherry (top two rows, depicted in red) or α-cateninΔVBS-mCherry, which lacks the vinculin binding domain (bottom two rows, depicted in red). In addition, cells express zebrafish vinculinA-GFP or vinculinB-GFP (both depicted in green) and were stained for F-actin (blue). Asterisks mark Focal Adhesions, White arrows mark Focal Adherens Junctions and Yellow Arrowheads mark Linear Adherens Junctions.

To study the localization of the two zebrafish vinculin isoforms as well as their interaction with α-catenin, we generated α-catenin-depleted MDCK epithelial cells stably expressing murine α-catenin or α-catenin-DVBS (a mutant α-catenin construct lacking the vinculin binding site [16,17]), in which we transiently expressed GFP-tagged versions of zebrafish vinculin A and B. In α-catenin rescued cells, both vinculin A and vinculin B localize to integrin-based Focal Adhesion structures, as well as to the punctate Focal Adherens Junctions as evidenced by colocalization with α-catenin (Fig 2 - top 2 rows, asterisks and yellow arrowheads respectively). Localization to Linear Adherens Junctions (white arrows) was also observable for both isoforms albeit with less intensity. This closely mimics the localization of chicken vinculin that has been extensively studied previously [16]. Curiously, the 822F residue in zebrafish vinculin B does not perturb its localization to cell-cell junctions. In addition, we can interfere specifically with vinculin A and B recruitment to cell-cell junctions using α-cateninΔVBS (Fig 2 - bottom). Here it is apparent that both vinculin A and vinculin B are now excluded from Focal Adherens Junctions and Linear Adherens Junctions (arrows), but are still present in Focal Adhesions (arrows). We can thus conclude that both zebrafish vinculin isoforms localize similarly to chicken vinculin. This strongly indicates that both *vcla* and *vclb* genes in zebrafish generate fully functional vinculin proteins.

### Vinculin A is not needed for early zebrafish development

**Fig 3:**
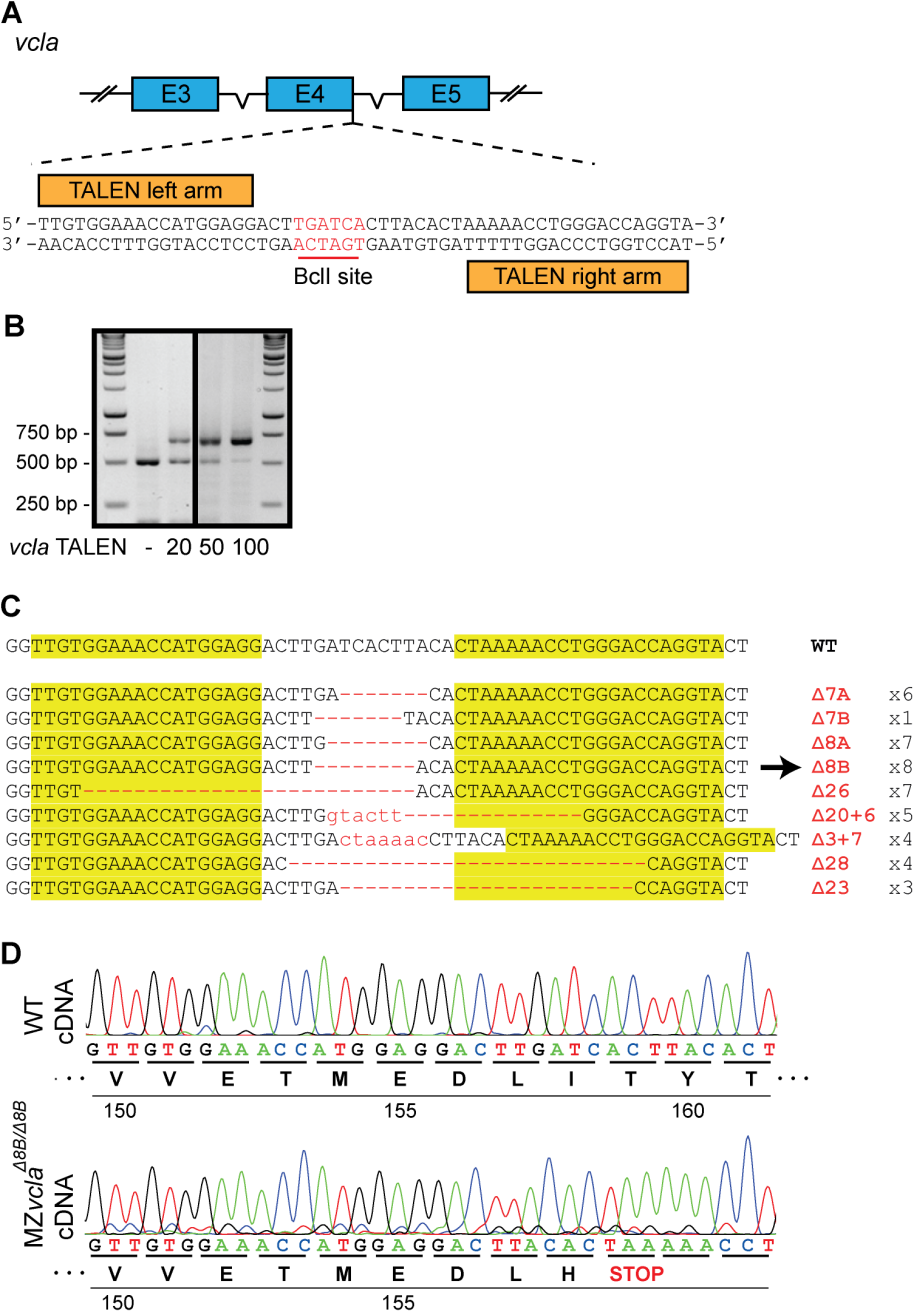
Generation of vinculin A-deficient zebrafish mutants using TALENs. (A) Schematic representation of the endogenous *vcla* locus targeted by TALEN gene editing technology at the exon4-intron4 boundary. The TALEN arms flank a BclI restriction enzyme recognition site (highlighted in red) used for screening mutant alleles through restriction fragment length polymorphism (RFLP) analysis. (B) RFLP analysis of embryos injected with increasing dosages of *vcla* TALEN mRNA. Uncleaved bands represent efficient TALEN activity. (C) Summary of the range of mutations found at the vcla TALEN target locus in the F1 offspring of *vcla* TALEN-injected fish. Arrow denotes the mutation of the *vcla* mutant used in further experiments. (D) Sequence chromatograms from cDNA of wild-type vinculin (top) and of the *vcla Δ8B* mutant allele (bottom).

To investigate the importance of vinculin during zebrafish morphogenesis, we first targeted the endogenous Vinculin A (*vcla*) locus in the exon 4-intron 4 boundary using the recently established TALEN gene editing technology [41,59] (Fig 3A). As the site contains a BclI restriction enzyme recognition sequence in the TALEN cleavage region, Restriction Fragment Length Polymorphism (RFLP) analysis could be utilized to assess the genotype of TALEN-injected embryos. The amplified target locus yields a PCR product of 683 bp, while BclI cleaved PCR product yields fragments of 504 bp and 179 bp (Fig 3B). Using PCR products from the genomic *vcla* locus of non-injected embryos results in efficient cleavage of the BclI site. In contrast, results from *vcla* TALEN-injected embryos also show additional uncleaved PCR product, indicating that the *vcla* TALEN is able to induce indel mutations at the target locus. Furthermore, increased dosages of injected v*cla* TALEN mRNA resulted in an increased amount of somatic mutations detected, as shown by decreasing amounts of BclI-digested PCR products of the amplified TALEN target site. All of 56 *vcla* TALEN-injected embryos which were screened by RFLP showed TALEN activity, even in the lowest dosage of injected *vcla* TALEN mRNA (S1 Fig). Thus, the *vcla* TALEN is able to efficiently induce mutations in the endogenous *vcla* locus.

The *vcla* TALEN-injected embryos were grown to adulthood and genotyped by fin clipping to identify potential founders. From embryos injected with 20 pg of *vcla* TALEN mRNA, 23 out of 40 (58%) fish grown to adulthood were found to be positive for somatic v*cla* mutations as assessed by RLFP. As expected, from embryos injected with higher dosages of *vcla* TALEN mRNA a higher percentage of fish with somatic mutations in the *vcla* locus were detected: 28 out of 32 (88%) for 50 pg of TALEN mRNA, and 23 out of 26 (88%) for 100 pg of TALEN mRNA (S2 Fig). Potential founders were outcrossed to wild-type fish, and the resulting offspring was screened for germline transmission of mutant alleles. From offspring of three individual crosses, a total of 100 embryos were genotyped of which 47 embryos were detected with a mutant *vcla* allele (S3 Fig). F1 offspring from positive *vcla* mutant founders were grown to adulthood, and subsequently genotyped by fin clipping. Several different indels in the endogenous *vcla* TALEN target locus were found, with some mutations occurring more frequently than others (Fig 3C). This suggests that TALEN activity at the *vcla* target locus can favor certain mutations over others. The v*cla* Δ8B mutation results in a frameshift that creates a premature stopcodon at amino acid position 160. Translation of this mRNA would result in a highly truncated protein, containing only the first helical domain and a small part of the second (Fig 1B). Such constructs generated from chicken vinculin have been tested in vitro and were found to be highly instable [47]. We therefore hypothesize that the resulting protein fragment lacks any functionality or interfering capacity. Thus, mutant *vcl*^*hu10818*^ will be regarded as a loss of function mutant and will be the main *vcla* mutant discussed further, unless otherwise specified.

To screen for potential phenotypes due to loss of *vcla*, the heterozygous *vcla* mutants were incrossed. The presence of homozygous *vcla* mutants was confirmed using the RFLP assay. However no major developmental phenotypes could be detected even after 5 dpf. To rule out possible effects of maternally contributed mRNA or protein of v*cla* to early development, the offspring was grown to adulthood, then incrossed to generate Maternal Zygotic (MZ) *vcla* embryos. All the *vcla* mutants described in the rest of this manuscript, will be the MZ *vcla* mutant. Surprisingly, these embryos also did not show gross developmental defects, and were fully viable. To investigate whether exon skipping or an alternative transcriptional start site would still generate a partial *vcla* gene product, we sequenced cDNA from MZ *vcla* mutants (Fig 3D). The sequencing results clearly show the expected 8 bp deletion leading to a premature stopcodon at position Y160 in both alleles, indicating that the presence of functional *vcla* protein is highly unlikely. Taken together, the results show that *vcla* is not essential for zebrafish development or adult life.

### Vinculin B-deficient embryos do not show defects during development

**Fig 4:**
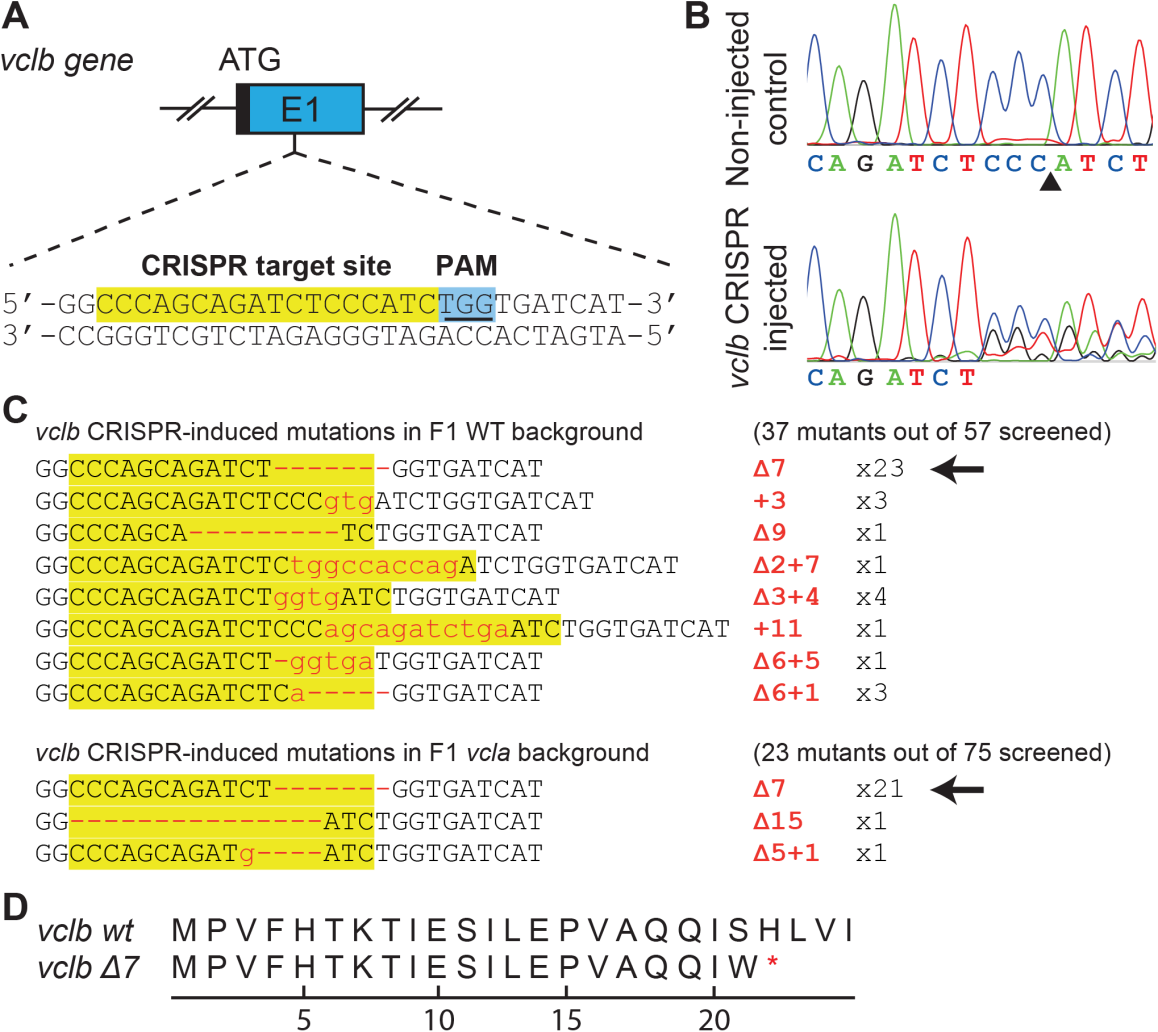
Generation of vinculin B-deficient zebrafish using CRISPR-Cas. (A) Schematic representation of the endogenous *vclb* locus showing the CRISPR guide RNA target site in exon 1. (B) Sequence chromatograms of genomic DNA from non-injected control (top) and *vclb* CRISPR-injected embryos. The arrowhead denotes the expected CRISPR cleavage site (3 nucleotides upstream of the PAM site). (C) Summary of mutations found in the F1 offspring of *vclb* CRISPR-injected fish of either a wild-type (top) or *vcla* mutant (bottom) background. Arrows denote the mutation of the *vclb* mutant used in further experiments. (D) Amino acid sequence of *vclb* in wild-type (top) and *vclb* mutant (bottom). Asterisk denotes the premature stop codon found in the vinculin B coding sequence of *vclb* mutants.

To study the effects of total loss of vinculin in zebrafish, we next sought to disrupt the *vclb* locus as well. To this end we used CRISPR-Cas gene editing technology [60], generating guide RNAs targeting exon 1 (Fig 4A). Injection of guide RNA together with Cas9 mRNA resulted in deletions at the *vclb* CRISPR target site, showing that the chosen guide RNA is able to efficiently cleave the endogenous *vclb* locus (Fig 4B). We injected the *vclb* guide RNAs together with Cas9 mRNA in embryos from both *vcla* wild-type and mutant background. The injected F0 embryos were then grown to adulthood, and outcrossed to wild-type and *vcla* mutants respectively. The genomic DNA of the resulting F1 generation was subsequently genotyped by fin clipping to identify potential founders harboring a single *vclb* mutation. Of F1 fish screened 65% (37 out of 57) with a wild type background and 30% (23 out of 75) with *vcla* mutant background showed germline mutations. While we found a range of different indels at the *vclb* locus, a 7 bp deletion was dominant in both lines (Fig 4C). This frameshift mutation generates a premature stopcodon at position 22 (Fig 4D) and is the *vclb* mutant allele we describe from here on, unless otherwise specified, and is designated as *vclb*^*hu11202*^. Thus, we have generated a *vclb* mutant which we can use in combination with our previously generated *vcla* mutant, to study complete loss of vinculin in zebrafish development.

### Zygotic loss of vinculin induces mild developmental defects during embryonic stages

**Fig 5:**
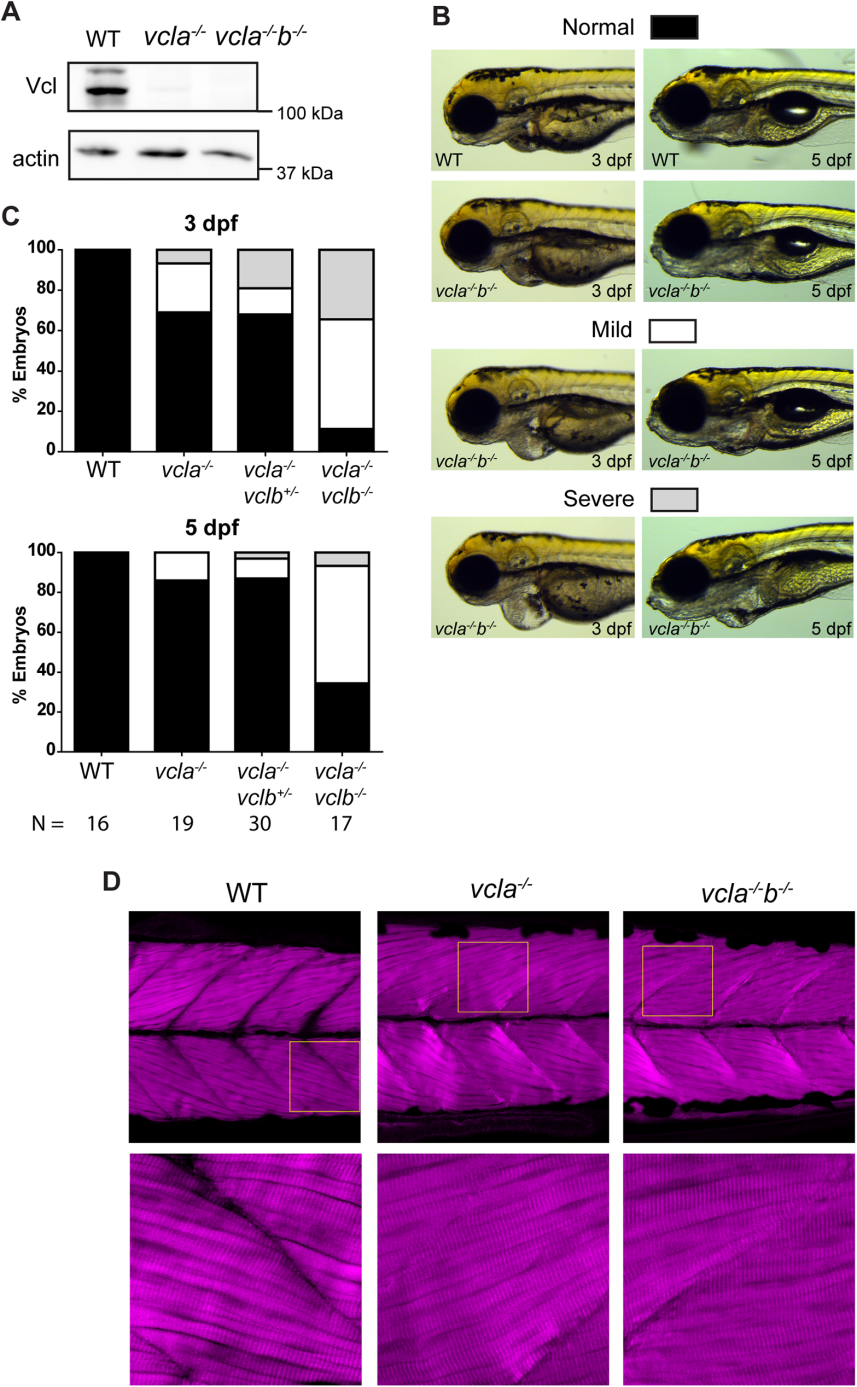
Cardiac and skeletal muscle phenotypes of vinculin-null mutants. (A) Western blot of lysates from the posterior half of WT, *vcla*^-/-^ and *vcla*^-/-^*vclb*^*-/-*^ embryos at 5 dpf, probed for vinculin and β-actin. (B) Some vinculin mutants show cardiac edema of which representative mild and severe cases are depicted. Wild type and *vcla*^-/-^*vclb*^*-/-*^ mutants at 3 dpf (left) and 5 dpf (right). For corresponding images of the other genotypes described in (C), see supplemental S6 Fig. (C) Quantification of the presence of cardiac edemas in offspring from a *vcla*^-/-^*vclb*^*+/-*^ incross. Classification as depicted in (B). Data was obtained from three independent experiments. WT embryos from an independent WT strain were analyzed as control from two independent experiments. Data is represented as mean ± s.e.m. A two-tailed paired student t-test was performed to compare the incidence of severe edemas between 3 dpf and 5 dpf within each genotype (see S7 Fig). (D) Immunostaining of actin in skeletal muscle of 5 dpf embryos. Images at the bottom are zoomed in parts of the upper images as indicated by the yellow squares (see S8 Fig for additional images and quantifications).

To study whether complete loss of vinculin would result in disturbed embryogenesis, we incrossed *vcla*^-/-^*vclb*^*+/-*^ zebrafish lines. Surprisingly, we could not detect any major developmental defect during early development, with vinculin-null embryos virtually indistinguishable from their siblings at 1 dpf (S4 Fig). We then continued observing the same embryos at 1 dpf, 3,5 dpf and 5 dpf. Out of 67 embryos observed, only 1 embryo was found dead at 5 dpf, indicating no major lethality during the first 5 days of embryogenesis. At 5 dpf, the embryos were cut in half and the anterior halves were used for genotyping while the posterior halves were used for westernblot analysis of vinculin expression. Mendelian inheritance of the mutant alleles was confirmed by the genotyping, as we indeed found ±25% of the F1 generation to be homozygous mutant for both vinculin genes (S2A Table). By westernblotting using an antibody that recognizes both zebrafish vinculin A and B proteins (S5 Fig) we detect a very strong reduction in vinculin protein levels in the *vcla*^-/-^ mutants, while there is no vinculin protein detectable in the *vcla*^-/-^*vclb*^*-/-*^ double mutants (Fig 5A). This result strongly indicates that mutation of the *vcla* gene leads to loss of most of the functional vinculin protein in zebrafish embryos and that this is not compensated for by increased expression of vinculin B. Moreover, the very low expression of vinculin B at day 5 indicates that indeed very little if any vinculin protein is present due to maternal contribution in the early stages of development of the *vcla*^-/-^*vclb*^*-/-*^ double mutants.

While no lethality was observed, we did notice that *vcla*^-/-^*vclb*^*-/-*^ double mutants began to develop cardiac edemas around 3 dpf that arbitrarily classified by eye in mild (54%) and severe (34%) cases (Fig 5B and 5C). In some of the most severe cases, a shorter body length and craniofacial defects were also observed, although these could also be observed in some of the *vcla*^-/-^*vclb*^*+/-*^ embryos (see S6 Fig for multiple images of all phenotypes). The chance of developing the cardiac phenotype seemed to be gene dose dependent, as only 13% of *vcla*^-/-^*vclb*^*+/-*^ mutants and 24% of *vcla* mutants developed mild cardiac edemas, while the incidence of severe edemas was also lower (19% and 17% for *vcla*^-/-^*vclb*^*+/-*^ and *vcla* mutants respectively) compared to *vcla*^-/-^*vclb*^*-/-*^ double mutants. At 5 dpf the majority of the cardiac edemas had disappeared in *vcla*^-/-^*vclb*^*+/-*^ and *vcla* mutants. Also in *vcla*^-/-^*vclb*^*-/-*^ mutants, cardiac edema was apparently transient as only 8% still showed severe edemas at 5 dpf while a third of the mutants now looked similar to wild-type (Fig 5C). Statistical testing did not show significance of the observed differences (S7 Fig). These results show that loss of vinculin leads to, potentially transient, cardiac edemas in a number of embryos that are not -ethal during embryonal stages.

Vinculin’s role in mechanotransduction suggests that tissues subjected to large mechanical forces, such as cardiac and skeletal muscles, are more likely to be affected by loss of vinculin than other tissues. Indeed, loss of vinculin in *C*. *elegans* [24], and modulation of vinculin functioning using truncated vinculin isoforms in *Drosophila* resulted in muscle defects [27]. However, when we inspected the morphology of skeletal muscle in *vcla*^-/-^*vclb*^*-/-*^ zebrafish embryos at 5 dpf we could not detect any major defect (Fig 5D). No large ultra-structural defects can be found, and individual muscle fibres in these mutant embryos are indistinguishable from wild-type tissue. We did observe some irregularities along the intersomitic boundaries in about half of the boundaries analyzed (S8 Fig), but since these were also found in wild-type samples with a similar frequency we attribute these to the sample preparation method used. Since no major developmental defects occurred in the vinculin-null background, embryos from *vcla*^-/-^*vclb*^*+/-*^ incrosses were grown to adulthood and genotyped by fin clipping at 10–12 weeks post fertilization. While one of our lines showed a high mortality rate (69%) during this time (104 dead out of 151), two other independent crossings had mortality rates of 37% and 26% (22 dead out of 59, and 70 dead out of 270 respectively) (S2 Table). Remarkably, during fin clipping no *vcla*^-/-^*vclb*^*-/-*^ fish were detected (172 adults screened out of three different pairings), while the remaining *vcla*^-/-^ and *vcla*^-/-^*vclb*^*+/-*^ siblings roughly show a Mendelian distribution. This result corroborates a recent study in which loss of *vclb* alone leads to lethality during juvenile stages [40]. Taken together, the data indicate that vinculin is not essential for early development of the zebrafish embryo.

## Discussion

In this study we show that zebrafish contain two homologous vinculin isoforms, of which the sequence of key functional domains is conserved with that of several higher vertebrate vinculin proteins. Loss of both zygotic *vcla* and maternal contribution of *vcla* did not result in notable defects, while the additional loss of zygotic *vclb* in this mutant background is lethal only after embryonic stages. These data indicate that embryonic development in zebrafish can occur without vinculin.

From the sequence analysis and localization, it stems that both vinculin isoforms are bona fide vinculin proteins. Both proteins are stable when expressed in cultured cells and localize similarly to endogenous or ectopically expressed chicken vinculin (Fig 2). The very high homology in regions of vinculin where key interactions for its functioning take place indicates that besides localization, also the main interactions are conserved (Fig 1C–1E), although differences cannot be excluded from sequence analysis alone. Differences in post-translational modifications that may affect vinculin’s functions can also not be excluded. Most of the known residues of phosphorylation (as indicated by phosphosite.org), whether or not functionally important, are conserved between zebrafish and higher vertebrate vinculins (not shown). There is, however, one notable exception. Recently, residue Y822 was found to be a determinant for vinculin localization and functioning specifically in cell-cell junctions [58]. The unphosphorylatable Y822F mutant was unable to localize in cadherin-based junctions and was unable to stabilize cell-cell adhesions due to reduced β-catenin binding. In our experiments, we found that the Y822F mutation is naturally present in zebrafish vinculin B, but did not impede its localization to cadherin junctions (Fig 2). Furthermore, only when perturbing the α-catenin-vinculin interaction in α-catenin-ΔVBS, which does not in any way affect vinculin or β-catenin, was vinculin excluded from junctions. So far, perturbation of cell-cell junction localization by the Y822F mutation was only shown in MCF10A cells, whereas in MDCK cells chicken vinculin Y822F localized normally (our unpublished data). This apparent discrepancy may arise due to different junction structures formed in different cell types. Alternatively, the fact that endogenous vinculin was depleted in experiments performed by Bays et al. but was still present in our cells, may have rescued the junctional localization of Y822F vinculin or zebrafish vinculin B. Indeed, vinculin proteins in solution have been shown to dimerize through their tail domains [61]. In conclusion, from the currently available data, both vinculin proteins in zebrafish appear to function comparably to the single vinculin protein present in higher vertebrates and can be regarded as functionally redundant.

Despite apparent conservation of vinculin, it should be noted that important differences do exist between cadherin adhesion systems in mammals versus zebrafish. This is exemplified by investigation of the zebrafish isoform of vinculin’s closest paralog, αE-catenin. This protein is also highly homologous (90% sequence identity) to its murine counterpart [62]. However, it is monomeric in solution and forms a complex with β-catenin and F-actin simultaneously in solution. This is a key difference with higher vertebrate homologues, which form dimers and cannot bind to β-catenin and F-actin simultaneously under these conditions. It is therefore anticipated that allosteric regulation of α-catenin is needed for it to be able to link the cadherin complex to actomyosin, become part of a force chain and function as a mechanosensor. The factor that would regulate this is so far elusive. It is important to note that force itself was found to increase the affinity of α-catenin for β-catenin in a typical example of catch-bond behavior [63]. Because these apparent differences in force-response exist for the interaction between higher vertebrate α-catenin and zebrafish α-catenin with F-actin, this could indicate that mechanical properties and regulations of the cadherin adhesion system have not evolved similarly between these branches. As vinculin is a mechano-responsive protein in cadherin junctions this could mean that this particular function may not be conserved either. So far, however, this is still speculative. *In vitro* biophysical experiments with zebrafish α-catenin are awaited to directly compare α-catenin-vinculin modules from zebrafish and higher vertebrates.

We employed both TALEN and CRISPR-Cas9 gene editing technologies for targeted disruption of the vinculin A and B loci. While the techniques have only recently been established, many groups have shown successful gene targeting in zebrafish [59,60], *Drosophila* [64] and even human material [65]. One drawback of this approach as opposed to forward-genetic screens is the unpredictability of the genetic lesion. We induced indel mutations using TALENs or CRISPR-Cas9 to generate premature stop codons, most likely leading to nonsense-mediated mRNA decay [66]. However, translation could be possible at potential alternative translation start sites located after the premature stop-codon [67]. For the *vcla* mutant allele we describe here, that could for instance result in a truncated vinculin protein starting at M167 in exon 5. Such a protein would be missing most of the D1 domain crucial for vinculin functioning as both talin and α-catenin bind this region. In contrast, for *vclb* a potential truncated protein could start at M26 in exon 1, leaving the possibility for a functional vinculin protein. However, since the double vinculin mutant is lethal after embryonal stages, just as the single *vclb* mutants recently described [40], it is unlikely that such a truncated protein is really expressed. Another often-cited problem inherent to these DNA-editing techniques is the possibility of off-target effects [68–71]. While we did not investigate the presence of off-target mutations, the fact that we only observe a (late) phenotype in double *vcla vclb* mutants but not in single *vcla* mutants argues against this possibility.

Whereas deletion of the vinculin A gene did not cause any observable phenotype, a previous study in which vinculin knockdown was achieved by morpholino injection in zebrafish embryos, induced severe defects in both cardiac and skeletal muscle at 3 dpf [34]. Moreover, the splice-site morpholino that was used, only targeted the zygotic vinculin A isoform, keeping maternally supplied mRNA and vinculin B intact. The discrepancy in phenotypes described between *vcla* morpholino knockdown in this study and loss of *vcla* in our MZ *vcla* mutant is in line with several reports which show that many genetic lesions induced by TALEN or CRISPR-Cas9 do not recapitulate previously reported morpholino phenotypes [72]. In these cases either the morpholino phenotype was regarded as an off-target effect [73], or genetic ablation of a gene caused upregulation of a related/redundant protein to maintain homeostasis, a process less prone to occur with morpholino knockdown [74]. While we cannot fully exclude the latter, the most likely explanation is that the reported effects of the used vinculin A morpholinos are non-specific. The only functional homolog of vinculin A that we could identify in the zebrafish genome is vinculin B. First of all, we did not find evidence for upregulated expression of vinculin B in the *vcla* mutants (Fig 5A). Secondly, in our MZ *vcla* Z *vclb* mutant, vinculin B cannot be upregulated in the zygote. If indeed vinculin A MO effects were specific, one would then expect to see these morpholino-induced phenotypes also in the context of our mutants. While we do see cardiac edemas in vinculin-null mutants, skeletal muscle tissue seems unaffected.

We did not observe any major morphological defects in MZ *vcla* Z *vclb* mutants during early development but these fish did not survive until adulthood. The recent study by Cheng et al. in which *vclb* alone was perturbed showed that vinculin B is essential for epicardial differentiation and proper development of coronary vessels, with loss of *vclb* leading to death at juvenile stages [40]. This likely explains why additional loss of *vclb* in our *vcla* mutants leads to lethality after early development. Nevertheless we found that a total loss of vinculin induces mild and transient cardiac edema in a subset of otherwise normal-looking fish, showing that vinculin is not essential for early zebrafish development, similar to *Drosophila* of which vinculin mutants are viable and fertile [25]. This contrasts vinculin depletion in mice, which is lethal during embryonic stages, leading to defects in neural tube closure, and other tissues such as heart [28]. Moreover, disrupting the mechanosensitive domain of α-catenin blocks vinculin recruitment to Focal Adherens Junctions (Fig 2), attenuates the force-induced reinforcement of cadherin adhesions in epithelial cell model systems, and alters junction dynamics [12,16]. This same disruption also induced severe migration defects of the lateral mesoderm during convergent extension movements in zebrafish [18]. Loss of vinculin was therefore expected by us to lead to morphogenetic defects in multiple tissues. It is possible that the maternal supply of vinculin B that we could not lose due to adult lethality prevented appearance of early defects. On the other hand, perturbation of other integrin adhesion proteins in zebrafish such as Integrin-linked kinase [33], integrin-α7β1 [33,75], α-actinin [76] and paxillin [77] all result in severe skeletal muscle defects at later stages of development, which is not apparent in our vinculin mutants (Fig 5D). In the case of paxillin, a major interaction partner of vinculin in focal adhesions, deficiency in mice leads to embryonic lethality after E9.5 due to truncation of the anterior-posterior axis as well as defects in heart and somite development [78], similar as seen in the vinculin-null mice [28]. In zebrafish, zygotic depletion of paxillin leads to severe cardiac edema formation, cranial edemas, overall shortening of the anterior-posterior axis and skeletal muscle detachment defects [77]. While we observe a milder cardiac edema formation in our vinculin mutants, it is surprising that we do not observe the other severe developmental defects.

Although the fact that cardiac edema is observed warrants future research into the details of cardiovascular developments in vinculin-deficient embryos, we conclude here that vinculin is not of broad importance for tissue morphogenesis in zebrafish embryos.

## Supporting information

S1 Fig *vcla* TALEN activity in embryos. RFLP analysis of embryos injected with *vcla* TALEN mRNA. The TALEN cleavage activity was checked at 24 hpf. NIC = Non-injected control. Uncleaved PCR products indicate the presence of indels at the TALEN target site.
(TIF)

S2 Fig Somatic *vcla* mutations in adults after TALEN injection. Embryos injected with *vcla* TALEN mRNA were grown to adulthood after which DNA was isolated from the fin to check for somatic *vcla* mutations and identify potential founders. TALEN = Sample from a TALEN-injected embryo confirmed for TALEN activity as positive control. NIC = Non-injected control. Uncleaved PCR products indicate the presence of indels at the TALEN target site.
(TIF)

S3 Fig Germline transmission of the *vcla* mutant gene. Mutant *vcla* founders were outcrossed to wild-type and the resulting offspring was checked for germline transmission of the mutant *vcla* gene. p = Sample from a TALEN-injected embryo confirmed for TALEN activity as positive control. n = Non-injected control. Uncleaved PCR products indicate the presence of indels at the TALEN target site.
(TIF)

S4 Fig Offspring from incross of *vcla*^-/-^*vclb*^*+/-*^at 1 dpf. (TIF)

S5 Fig Western blot of vinculin A and B in mammalian cells. HEK293T cells were transfected with zebrafish vinculin A-GFP or vinculin B-GFP, lysed and immunoblotted as indicated.
(TIF)

S6 Fig Pericardial edema in vinculin mutants at 3 dpf and 5 dpf. Pericardial edemas of embryos of the different vinculin genotypes were categorized by eye into normal, mild and severe on 3 and 5 dpf from three independent experiments.
(TIF)

S7 Fig Comparison of cardiac edema of vinculin mutants between 3 dpf and 5 dpf. A two-tailed Student’s t-test was performed between the incidences of severe cardiac edema between 3 dpf and 5 dpf. Data is represented as mean ± s.e.m. from three independent experiments.
(TIF)

S8 Fig Skeletal muscle of vinculin mutants at 5 dpf. Representative images of skeletal muscle samples of wild-type control (A), *vcla* (B) and *vcla/b* double mutants (C) stained with phalloidin were analyzed at the intersomitic boundaries (bottom row). (D) quantification of the observed irregularities at the intersomitic boundaries. Data was obtained from two independent experiments.
(TIF)

S1 Table Identity matrix of vinculin isoforms. Identity matrix (in %) of the comparison between vinculin protein sequences of common model organisms. The matrix is based on multiple sequence alignment using Clustal Omega.
(PDF)

S2 Table Genotyping and mortality of offspring from *vcla*^-/-^*vclb*^*+/-*^ incrosses. (A) Embryos were observed from 1 dpf until 5 dpf, after which their genotype was assessed. Data was obtained from three independent experiments (B) Embryos were counted at 5 dpf and grown to adulthood. The resulting adult fish were again counted, and subsequently genotyped at 10–12 weeks post fertilization. Data was obtained from three independent experiments.
(DOCX)

S1 Text Multiple sequence alignment of vinculin proteins. Multiple sequence alignment of vinculin protein sequences from common model organisms using Clustal Omega. Stars represent complete conservation of the amino acid residue across all sequences. Dots and colons represent almost complete conservation, with differences in one or two residues respectively. The Used NCBI reference sequences: *Drosophila* (NP_476820.1), *C*. *elegans* (NP_501104.2), Human (NP_003364.1), Mouse (NP_033528.3), Chicken (NP_990772.1), *Xenopus* (NP_001090722.1). Zebrafish vinculin sequences were determined from our own cDNA clones.
(DOCX)

S2 Text Signed Arrive checklist. (PDF)
