# Supplementary figures and images for: Zygotic vinculin is not essential for embryonic development in zebrafish
Source: PLoS One. 2017 Aug 2;12(8):e0182278. doi: 10.1371/journal.pone.0182278 (PMC5540497; doi:10.1371/journal.pone.0182278)

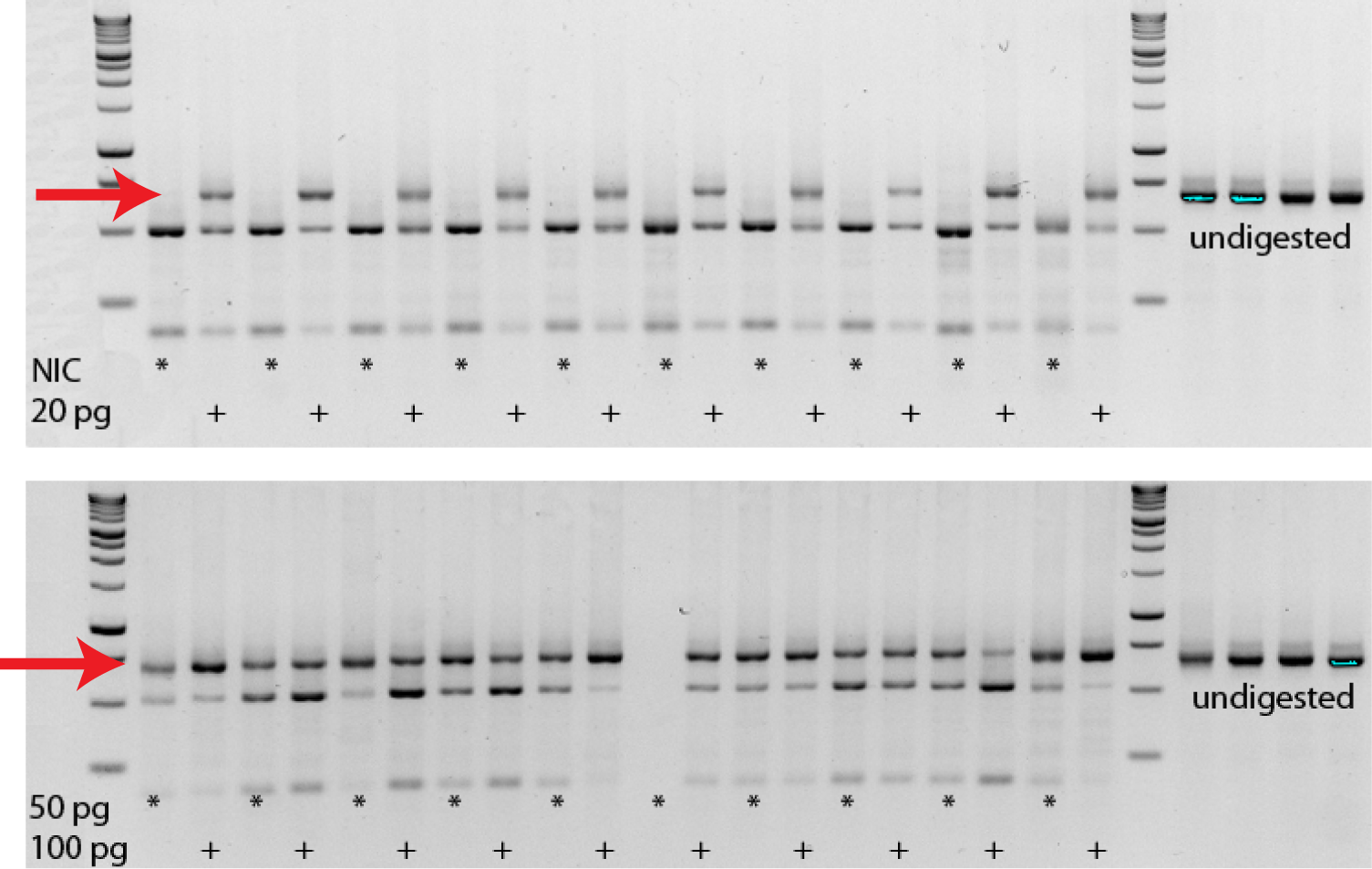

Supplement: S1 Fig — RFLP analysis of embryos injected with vcla TALEN mRNA. The TALEN cleavage activity was checked at 24 hpf. NIC = Non-injected control. Uncleaved PCR products indicate the presence of indels at the TALEN target site. (TIF) [file pone.0182278.s001.tif]

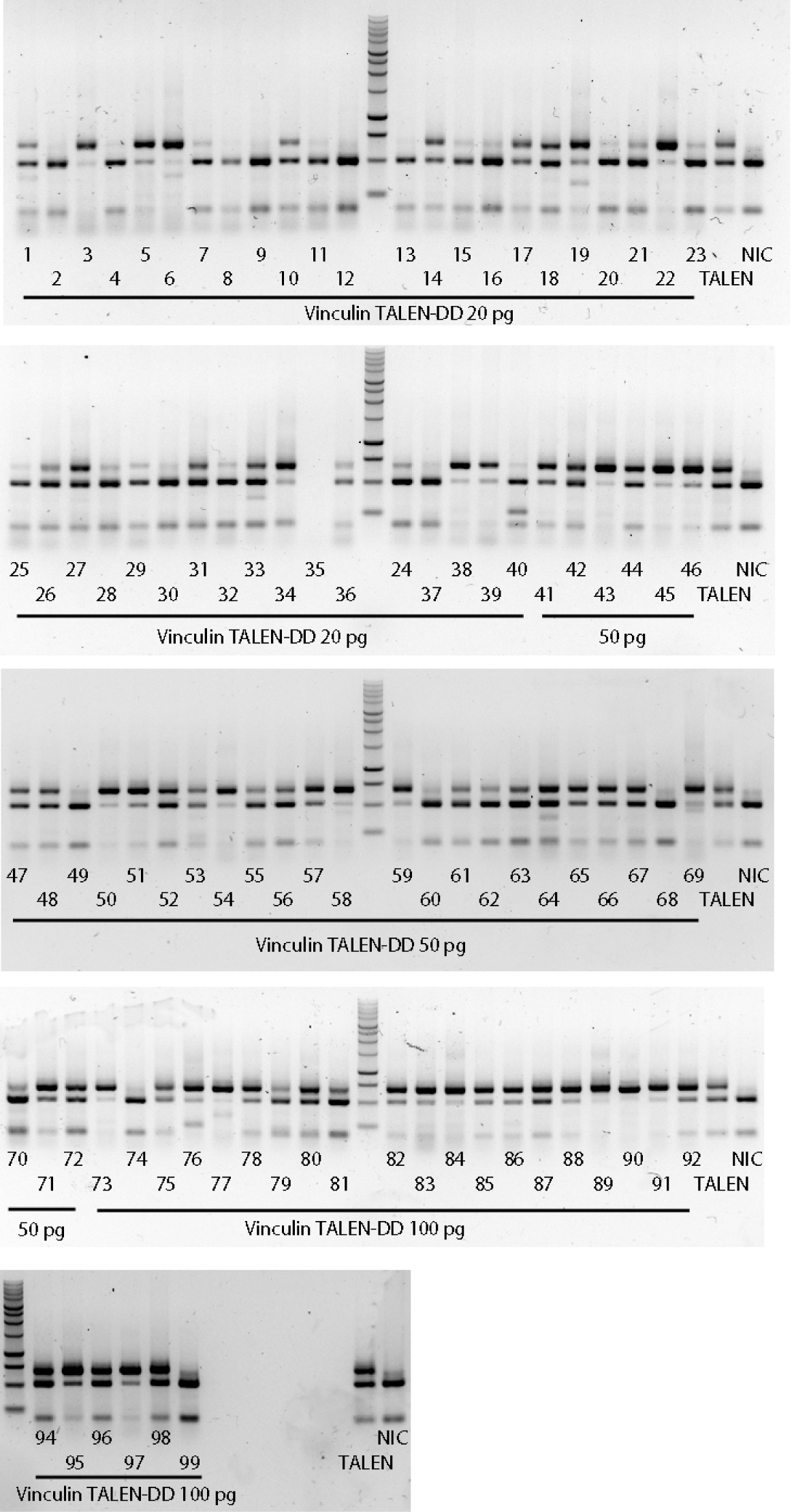

Supplement: S2 Fig — Embryos injected with vcla TALEN mRNA were grown to adulthood after which DNA was isolated from the fin to check for somatic vcla mutations and identify potential founders. TALEN = Sample from a TALEN-injected embryo confirmed for TALEN activity as positive control. NIC = Non-injected control. Uncleaved PCR products indicate the presence of indels at the TALEN target site. (TIF) [file pone.0182278.s002.tif]

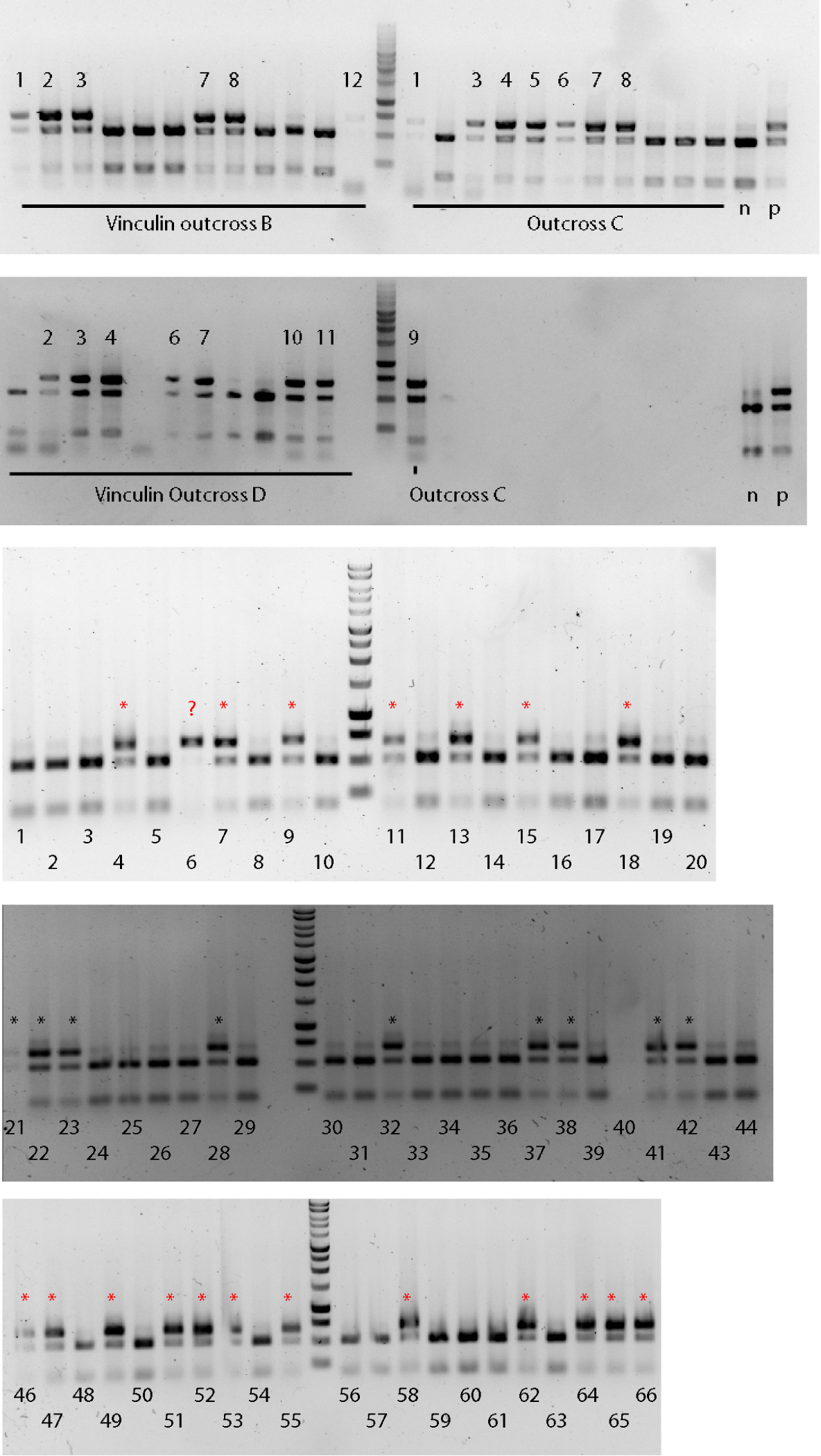

Supplement: S3 Fig — Mutant vcla founders were outcrossed to wild-type and the resulting offspring was checked for germline transmission of the mutant vcla gene. p = Sample from a TALEN-injected embryo confirmed for TALEN activity as positive control. n = Non-injected control. Uncleaved PCR products indicate the presence of indels at the TALEN target site. (TIF) [file pone.0182278.s003.tif]

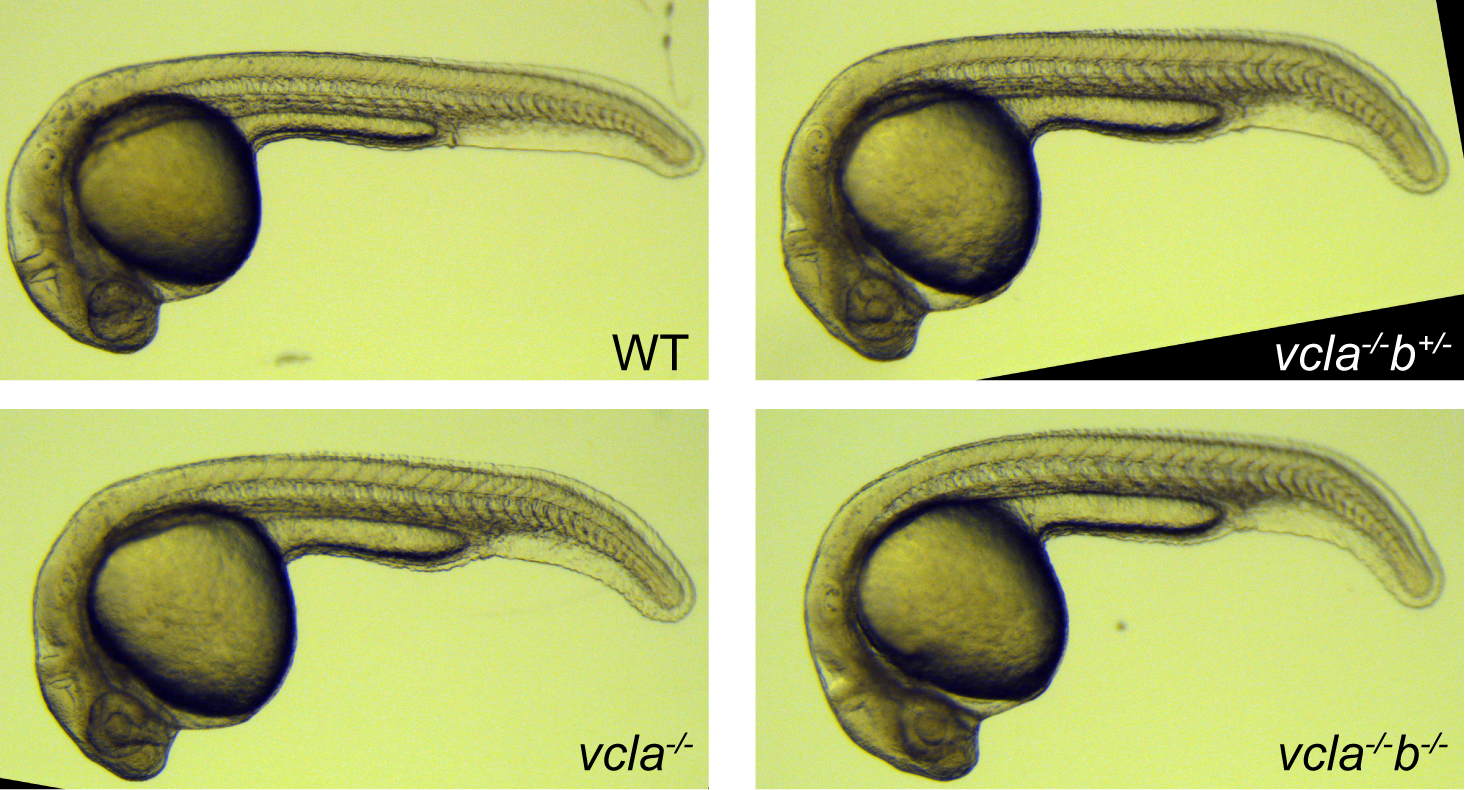

Supplement: S4 Fig — (TIF) [file pone.0182278.s004.tif]

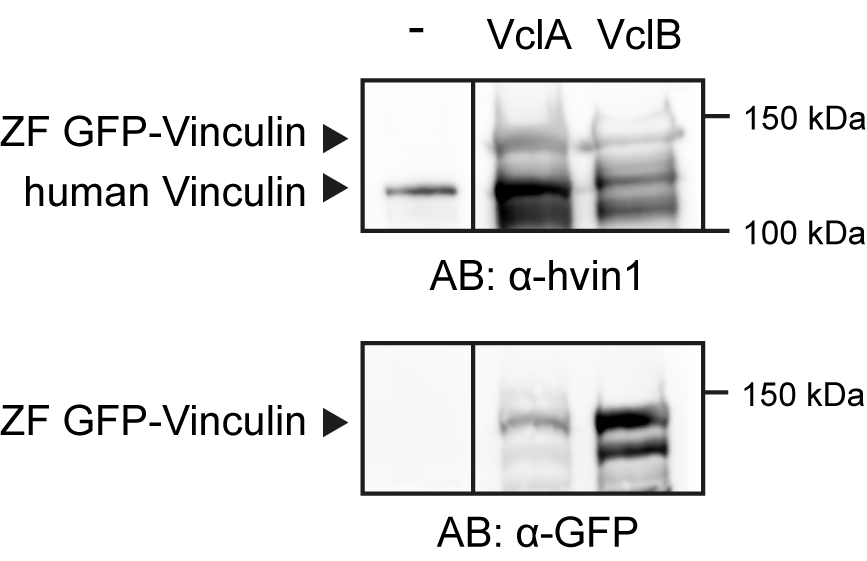

Supplement: S5 Fig — HEK293T cells were transfected with zebrafish vinculin A-GFP or vinculin B-GFP, lysed and immunoblotted as indicated. (TIF) [file pone.0182278.s005.tif]

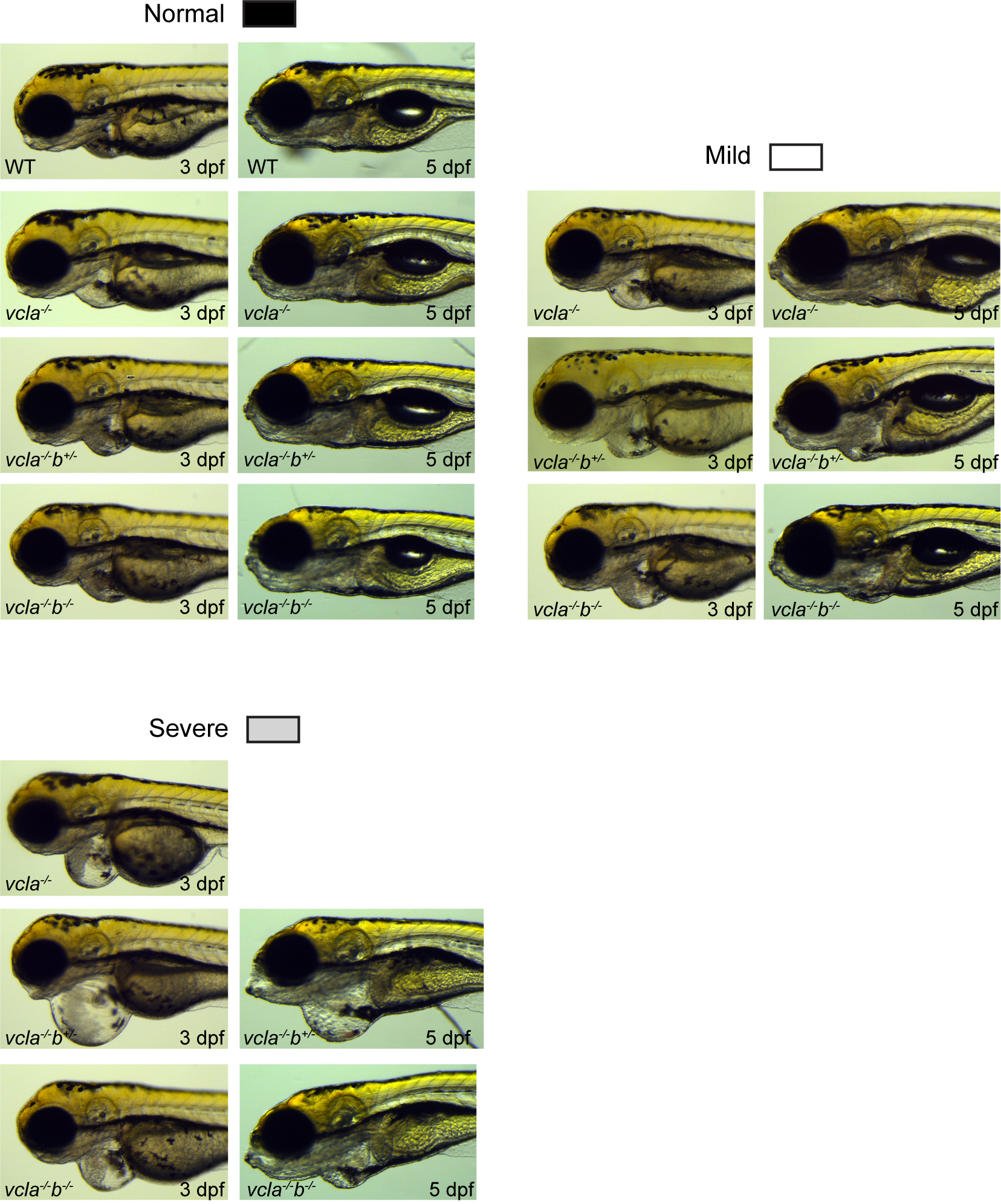

Supplement: S6 Fig — Pericardial edemas of embryos of the different vinculin genotypes were categorized by eye into normal, mild and severe on 3 and 5 dpf from three independent experiments. (TIF) [file pone.0182278.s006.tif]

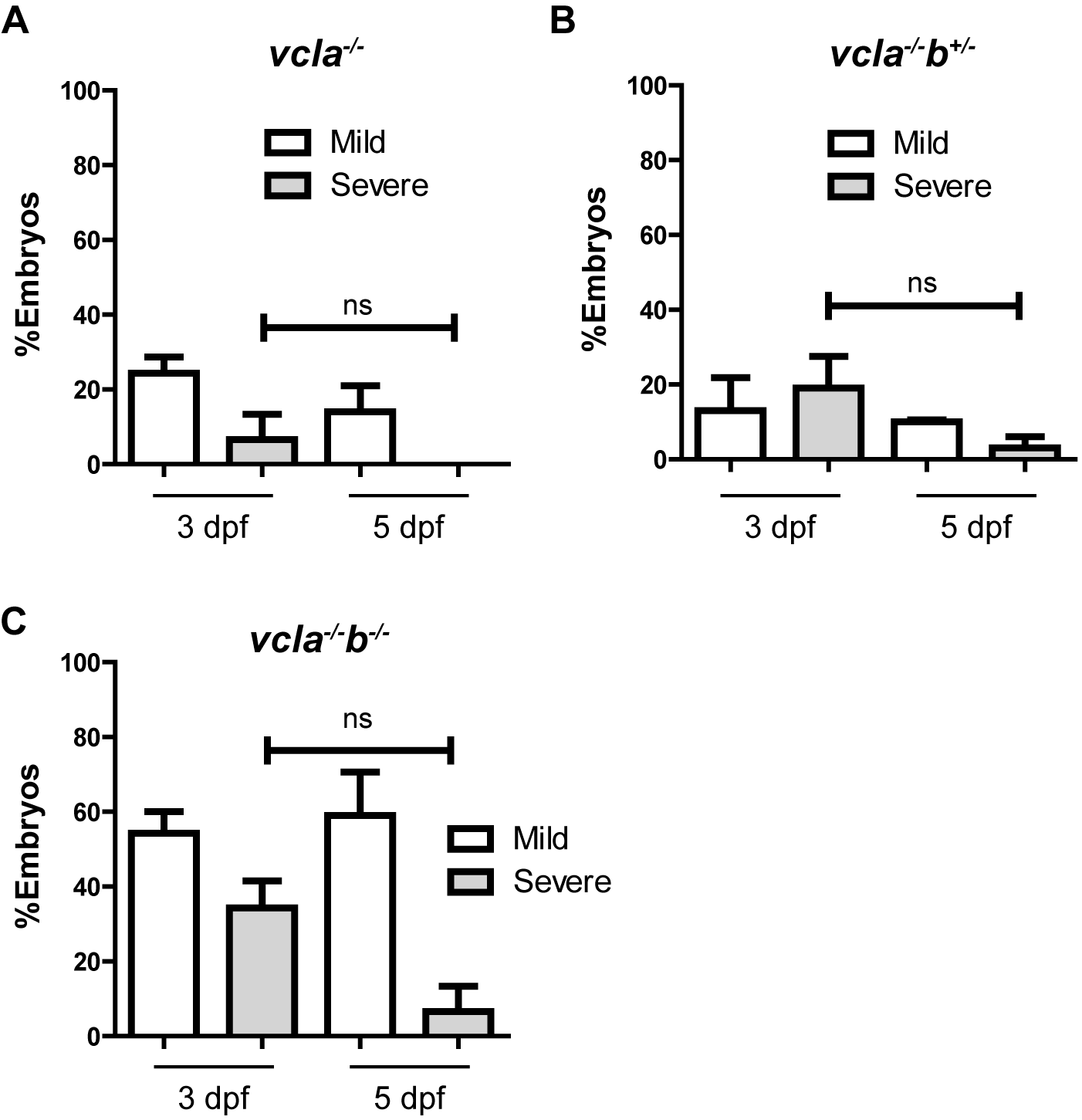

Supplement: S7 Fig — A two-tailed Student’s t-test was performed between the incidences of severe cardiac edema between 3 dpf and 5 dpf. Data is represented as mean ± s.e.m. from three independent experiments. (TIF) [file pone.0182278.s007.tif]

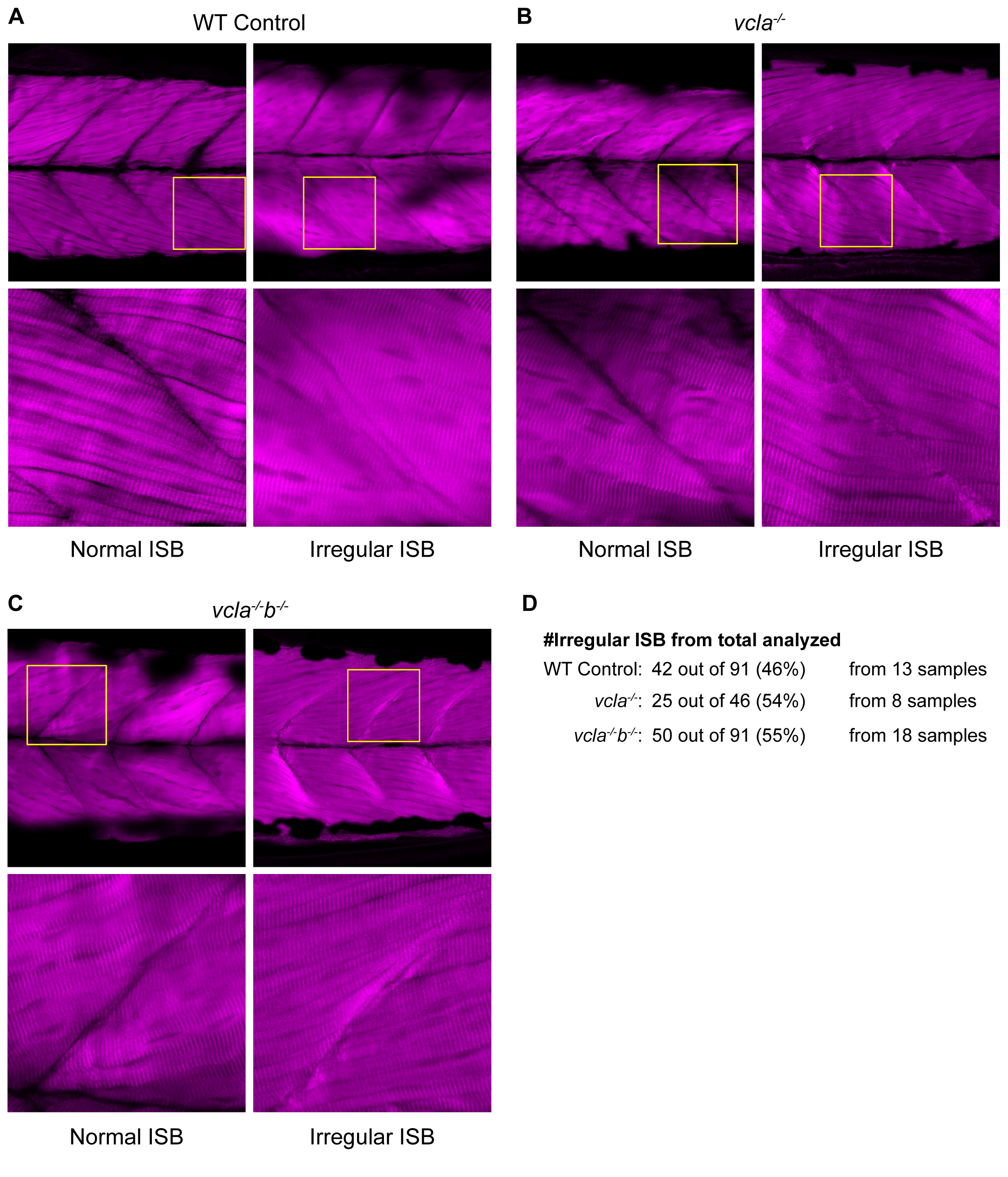

Supplement: S8 Fig — Representative images of skeletal muscle samples of wild-type control (A), vcla (B) and vcla/b double mutants (C) stained with phalloidin were analyzed at the intersomitic boundaries (bottom row). (D) quantification of the observed irregularities at the intersomitic boundaries. Data was obtained from two independent experiments. (TIF) [file pone.0182278.s008.tif]
