# Supplementary material for: Zygotic vinculin is not essential for embryonic development in zebrafish
Source: PLoS One. 2017 Aug 2;12(8):e0182278. doi: 10.1371/journal.pone.0182278 (PMC5540497; doi:10.1371/journal.pone.0182278)
Supplement: S1 Table — Identity matrix (in %) of the comparison between vinculin protein sequences of common model organisms. The matrix is based on multiple sequence alignment using Clustal Omega. (PDF) [file pone.0182278.s009.pdf]

Zebrafish_VclB								
Zebrafish_VclA	86							
Xenopus_Vcl	85	86						
Human_Vcl	86	87	90					
Mouse_Vcl	86	87	90	99				
Chicken_Vcl	86	88	90	95	95			
Drosophila_Vcl	47	48	47	48	48	48		
C. elegans_Vcl	46	45	45	46	46	46	51	

S1 Table. Identity Matrix of vinculin isoforms

Identity matrix (in %) of the comparison between vinculin protein sequences of common model organisms. The matrix is based on multiple sequence alignment using Clustal Omega.
