# Supplementary material for: Zygotic vinculin is not essential for embryonic development in zebrafish
Source: PLoS One. 2017 Aug 2;12(8):e0182278. doi: 10.1371/journal.pone.0182278 (PMC5540497; doi:10.1371/journal.pone.0182278)
Supplement: S2 Table — (A) Embryos were observed from 1 dpf until 5 dpf, after which their genotype was assessed. Data was obtained from three independent experiments (B) Embryos were counted at 5 dpf and grown to adulthood. The resulting adult fish were again counted, and subsequently genotyped at 10–12 weeks post fertilization. Data was obtained from three independent experiments. (DOCX) [file pone.0182278.s010.docx]

**A**

|  | **Total** | **VclA** | **VclA-Bhet** | **VclAB DKO** |  | **VclA** | **VclA-Bhet** | **VclAB DKO** |
| --- | --- | --- | --- | --- | --- | --- | --- | --- |
| **Incross 1** | 21 | 5 | 11 | 5 |  | 24% | 52% | 24% |
| **Incross 2** | 21 | 5 | 9 | 7 |  | 24% | 43% | 33% |
| **Incross 3** | 24 | 9 | 10 | 5 |  | 38% | 42% | 21% |
| **SUM** | **66** | **19** | **30** | **17** | **AVERAGE** | **28%** | **46%** | **26%** |

**B**

|  |  |  |  | **Genotype at 12 weeks** | | |  | **Genotype at 12 weeks** | | |
| --- | --- | --- | --- | --- | --- | --- | --- | --- | --- | --- |
| **#Embryos at 5 dpf** | **#Fish at 12 weeks** | **Mortality** |  | **VclA** | **VclA-Bhet** | **VclAB DKO** |  | **VclA** | **VclA-Bhet** | **VclAB DKO** |
| 151 | 47 | 69% |  | 12 | 34 | 0 |  | 26% | 72% | 0% |
| 59 | 37 | 37% |  | 11 | 25 | 0 |  | 30% | 68% | 0% |
| 270 | 200* | 26% |  | 27* | 61* | 0 |  | 31% | 69% | 0% |

*Only assayed 88 fish for genotyping

**S2 Table. Genotyping and mortality of offspring from** ***vcla*^-/-^*vclb^+/-^* incrosses**

(A) Embryos were observed from 1 dpf until 5 dpf, after which their genotype was assessed. Data was obtained from three independent experiments. (B) Embryos were counted at 5 dpf and grown to adulthood. The resulting adult fish were again counted, and subsequently genotyped at 10-12 weeks post fertilization. Data was obtained from three independent experiments.
