# Supplementary material for: Zygotic vinculin is not essential for embryonic development in zebrafish
Source: PLoS One. 2017 Aug 2;12(8):e0182278. doi: 10.1371/journal.pone.0182278 (PMC5540497; doi:10.1371/journal.pone.0182278)
Supplement: S1 Text — Multiple sequence alignment of vinculin protein sequences from common model organisms using Clustal Omega. Stars represent complete conservation of the amino acid residue across all sequences. Dots and colons represent almost complete conservation, with differences in one or two residues respectively. The Used NCBI reference sequences: Drosophila (NP_476820.1), C. elegans (NP_501104.2), Human (NP_003364.1), Mouse (NP_033528.3), Chicken (NP_990772.1), Xenopus (NP_001090722.1). Zebrafish vinculin sequences were determined from our own cDNA clones. (DOCX) [file pone.0182278.s011.docx]

Zebrafish_VclB MPVFHTKTIESILEPVAQQISHLVIMHEEGEVDGKAIPDLSAPVLAVQAAVSNLVRVGKE 60

Zebrafish_VclA MPVFHTKTIESILEPVAQQISHLVIMHEEGEVDGKAIPDLTSPVAAVQAAVSNLVRVGKE 60

Xenopus_Vcl MPVFHTKTIESILEPVAQQISHLVIMHEEGEVDGKAIPELTAPVAAVQAAVSNLVRVGKE 60

Human_Vcl MPVFHTRTIESILEPVAQQISHLVIMHEEGEVDGKAIPDLTAPVAAVQAAVSNLVRVGKE 60

Mouse_Vcl MPVFHTRTIESILEPVAQQISHLVIMHEEGEVDGKAIPDLTAPVAAVQAAVSNLVRVGKE 60

Chicken_Vcl MPVFHTRTIESILEPVAQQISHLVIMHEEGEVDGKAIPDLTAPVSAVQAAVSNLVRVGKE 60

Drosophila_Vcl MPVFHTKTIESILDPVAQQVSRLVILHEEAE-DGNAMPDLSRPVQVVSAAVANLVKVGRD 59

CElegans_Vcl MPVFHTKTIENILEPVAQQVSRLVILHEEAN-DGNAMPDLTGPVGMVSRAVGNLIQVGYD 59

******:***.**:*****:*:***:***.: **:*:*:*: ** *. **.**::** :

Zebrafish_VclB TVQTTEDAIMRRDMPPAFIKVENACTKLVQAAQMLKADPYSVPARDYLIDGSRGILSGTS 120

Zebrafish_VclA TVQTTEDKIMKRDMPSAFIKVENACAKLVEAAQMLRTDPYSVPARDYLIDGSRGILSGTS 120

Xenopus_Vcl TVQTTEDQIMKRDMPPAFIKVENACAKLVQAAQMLHADPYSVPARDYLIDGSRGILSGTS 120

Human_Vcl TVQTTEDQILKRDMPPAFIKVENACTKLVQAAQMLQSDPYSVPARDYLIDGSRGILSGTS 120

Mouse_Vcl TVQTTEDQILKRDMPPAFIKVENACTKLVQAAQMLQSDPYSVPARDYLIDGSRGILSGTS 120

Chicken_Vcl TVQTTEDQILKRDMPPAFIKVENACTKLVRAAQMLQADPYSVPARDYLIDGSRGILSGTS 120

Drosophila_Vcl TINSSDDKILRQDMPSALHRVEGASQLLEEASDMLRSDPYSGPARKKLIEGSRGILQGTS 119

CElegans_Vcl TCDHSDDRILQQDMPPALQRVEGSSKLLEESSYSLKHDPYSVPARKKLIDGARGILQGTS 119

* : ::* *:::*** *: :** :. * .:: *: **** ***. **:*:****.***

Zebrafish_VclB DLLLTFDEAEVRKIIRVCKGILEYLTVAEVVESMEDLITYTKNLGPGMTKMAKMIDERQQ 180

Zebrafish_VclA DLLLTFDEAEVRKIIRVCKGILEYLTVAEVVETMEDLITYTKNLGPGMTKMAKMIDERQQ 180

Xenopus_Vcl DLLLTFDEAEVRKIIRVCKGILEYLTVAEVVESMEDLVTYTKNLGPGMTKMAKMIDERQQ 180

Human_Vcl DLLLTFDEAEVRKIIRVCKGILEYLTVAEVVETMEDLVTYTKNLGPGMTKMAKMIDERQQ 180

Mouse_Vcl DLLLTFDEAEVRKIIRVCKGILEYLTVAEVVETMEDLVTYTKNLGPGMTKMAKMIDERQQ 180

Chicken_Vcl DLLLTFDEAEVRKIIRVCKGILEYLTVAEVVETMEDLVTYTKNLGPGMTKMAKMIDERQQ 180

Drosophila_Vcl SLLLCFDESEVRKIIQECKRVLDYLAVAEVINTMEQLVQFLKDLSPCLSKVHREVGAREK 179

CElegans_Vcl ALLLCFDESEVRKIIRVCRKANDYVAVSEVIESMADLQQFVKDISPVLHDVTNDVNLRQQ 179

*** ***:******: *: :*::*:**:::* :* : *::.* : .: . : *::

Zebrafish_VclB ELTHQEHRVMLVNSMNTVKELLPVLISGIKIFVTTRTSQ-GKGVEEALKNRNFTVEKMNT 239

Zebrafish_VclA ELTHQEHRVMLVTSMNTVKELLPVLISAIKIFVTTKCTK-SHGVEEALKNRNYTFDKMTA 239

Xenopus_Vcl ELTHQEHRVMLVNSMNTVKDLLPVLISAMKIFVTTKNSR-SQGIEEALKNRNFTVEKMSA 239

Human_Vcl ELTHQEHRVMLVNSMNTVKELLPVLISAMKIFVTTKNSK-NQGIEEALKNRNFTVEKMSA 239

Mouse_Vcl ELTHQEHRVMLVNSMNTVKELLPVLISAMKIFVTTKNSK-NQGIEEALKNRNFTVEKMSA 239

Chicken_Vcl ELTHQEHRVMLVNSMNTVKELLPVLISAMKIFVTTKNTK-SQGIEEALKNRNFTVEKMSA 239

Drosophila_Vcl ELTHQVHSEILVRCLEQVKTLAPILICSMKVYIHIVEQQ-GRGAEEAAENRNYLAARMSD 238

CElegans_Vcl ELTHQVHREILIRCMDSIKVIAPILICSMKTSIELGTPHPRQGHAEAIANRNFMSQRMTE 239

***** * :*: .:: :* : *:**..:* : : :* ** ***: :*.

Zebrafish_VclB EINEIIRVLQLTSWDEDAWAN-KDTEAMKRALALIDSKMAQAKNWLRDPQGQPGGPGEQA 298

Zebrafish_VclA EINEIIRVLQLTSWDEDAWANKKDTEAMKRALALIESKMGQAKGWLRDPNALPGDPGEHA 299

Xenopus_Vcl EINEIIRVLQLTSWDEDAWAS-KDTEAMKRALALIDSKINQAKGWLRDPNAPPGDVGEQA 298

Human_Vcl EINEIIRVLQLTSWDEDAWAS-KDTEAMKRALASIDSKLNQAKGWLRDPSASPGDAGEQA 298

Mouse_Vcl EINEIIRVLQLTSWDEDAWAS-KDTEAMKRALASIDSKLNQAKGWLRDPNASPGDAGEQA 298

Chicken_Vcl EINEIIRVLQLTSWDEDAWAS-KDTEAMKRALALIDSKMNQAKGWLRDPNAPPGDAGEQA 298

Drosophila_Vcl ELQEIIRVLQLTTYDEDTSEL-DNLTVLKKLSNAISNKMEQANEWLSNPYALRGGVGEKA 297

CElegans_Vcl EMNEIIRVLQLTTYDEDEWDA-DNVTVMRKALSAAKSLLTAALDWLADPHARSGAVGEKA 298

*::*********::*** .: .::: .. : * ** :* . * **:*

Zebrafish_VclB IRQILDEAEKVGELCAGKERRDIVGTAKTLGQLTEQVSDLRARGQGANPVAMQKAQQVSQ 358

Zebrafish_VclA LRQILDEAGKVGELCAGKERREILGTAKTLGQMTDQVSDVRARGQGATPMGMQKAQQVAQ 359

Xenopus_Vcl VRQILDEAGKVGELCAGTERKDILGICRTLGQMTDQVSDLRARGQGATPIAMQKAQQVSQ 358

Human_Vcl IRQILDEAGKVGELCAGKERREILGTCKMLGQMTDQVADLRARGQGSSPVAMQKAQQVSQ 358

Mouse_Vcl IRQILDEAGKVGELCAGKERREILGTCKMLGQMTDQVADLRARGQGASPVAMQKAQQVSQ 358

Chicken_Vcl IRQILDEAGKAGELCAGKERREILGTCKTLGQMTDQLADLRARGQGATPMAMQKAQQVSQ 358

Drosophila_Vcl LRQVIDNATEISERCLPQDSYPIRKLADEVTAMANTLCELRQEGKGQSPQAE----SLVR 353

CElegans_Vcl IRRICEYADRISARALPEDAQSIKRSIFEITSFTDELCNLRNNGQPDRENLAA---QTAR 355

:*:: : * . . . : * : ::: :.::* .*: . :

Zebrafish_VclB GLDVLTGKVENAARKLEAMTGSKQAIAKRIDAAQSWLADPHSGPEGEENIRALLGEARKI 418

Zebrafish_VclA GLDILVGKVENAARKLEALTNAKQAIAKRIDNAQSWLADPNGGPEGEENIRALLAEAKRI 419

Xenopus_Vcl GLDVLTSKVKNAAHKLEALTNSKQAIGKKIDAAQSWLADPNGGPEGEENIRTILAEAKKI 418

Human_Vcl GLDVLTAKVENAARKLEAMTNSKQSIAKKIDAAQNWLADPNGGPEGEEQIRGALAEARKI 418

Mouse_Vcl GLDVLTAKVENAARKLEAMTNSKQSIAKKIDAAQNWLADPNGGPEGEEQIRGALAEARKI 418

Chicken_Vcl GLDLLTAKVENAARKLEAMTNSKQAIAKKIDAAQNWLADPNGGSEGEEHIRGIMSEARKV 418

Drosophila_Vcl GIRDRMGE---------------------------------------L--KSL------- 365

CElegans_Vcl RLKDLVGS---------------------------------------QNSSGL------- 369

: ..

Zebrafish_VclB ADLCEDPKEREDILRSMSEIAALSGKLAELKRAGKGDTPEARALAKQIATALQNLQSKTS 478

Zebrafish_VclA ADLCEDPKERDDILRSIGEIAGLTARLVELRRIGKGDTPEARALAKQIGTALQNLQAKTN 479

Xenopus_Vcl ADLCEDPKDKEDILRSLGEIAALTAKLTDLRRQGKGDSHEARALAKQIATSLQNLQTKVN 478

Human_Vcl AELCDDPKERDDILRSLGEISALTSKLADLRRQGKGDSPEARALAKQVATALQNLQTKTN 478

Mouse_Vcl AELCDDPKERDDILRSLGEIAALTSKLGDLRRQGKGDSPEARALAKQVATALQNLQTKTN 478

Chicken_Vcl AELCEEPKERDDILRSLGEISALTAKLSDLRRHGKGDSPEARALAKQIATSLQNLQSKTN 478

Drosophila_Vcl -----------------------------------------------VHQAVLGVD---- 374

CElegans_Vcl -----------------------------------------------MGDALQNAQ---- 378

: :: :

Zebrafish_VclB KAVANTRPAKAAVHLAGKMEQAGRWIDNPTLDDSGVGQAAIRGLLAEGRRLANALPAAQR 538

Zebrafish_VclA RAVANMRPAKAAVTLEGKMEQALRWINNPGVDDHGVGQAAIRGLIAEGRRLASSLPGPYR 539

Xenopus_Vcl RAVANSRPVKAAVNMEGKVEQAQRWIDNPSVDDKGVGQAAIRGLVAEGRRLANSMIGPFR 538

Human_Vcl RAVANSRPAKAAVHLEGKIEQAQRWIDNPTVDDRGVGQAAIRGLVAEGHRLANVMMGPYR 538

Mouse_Vcl RAVANSRPAKAAVHLEGKIEQAQRWIDNPTVDDRGVGQAAIRGLVAEGHRLANVMMGPYR 538

Chicken_Vcl RAVANTRPVKAAVHLEGKIEQAQRWIDNPTVDDRGVGQAAIRGLVAEGRRLANVMMGPYR 538

Drosophila_Vcl ----KAGVQQTAHTIQGRLEQAVKWLQHPEINDGGLGERAINLIVEEGRKVAEGCPGHQK 430

CElegans_Vcl ----RHGGANPAHTAAGRLEQALRWLDNPGLDDGGLGLQALRLLTADARKLADRLNPQDR 434

. : * *::*** :*::.* ::* *:* *:. : :.:::*. :

Zebrafish_VclB QELLGKCEQVEHLMAQLAELAARGEGDSPQARAIAQQLQHTLKELEGKMQDAMTQEVSDI 598

Zebrafish_VclA QELLAKCEQVEQLMMQLADLAARGEGESPQARAVAAHLLEAIKDLKAKMQEAMTQEVSDV 599

Xenopus_Vcl QDMMAKCDRVEQLAGQLAELALRGEGDTPLAQAVAAQLQEALKDLKGKMQEAMTQEVSDV 598

Human_Vcl QDLLAKCDRVDQLTAQLADLAARGEGESPQARALASQLQDSLKDLKARMQEAMTQEVSDV 598

Mouse_Vcl QDLLAKCDRVDQLTAQLADLAARGEGESPQARALASQLQDSLKDLKAQMQEAMTQEVSDV 598

Chicken_Vcl QDLLAKCDRVDQLAAQLADLAARGEGESPQARAIAAQLQDSLKDLKARMQEAMTQEVSDV 598

Drosophila_Vcl AEIQQLCDEVERLK-------RQAAGSGPAAKQAAKQLTQKLYELKAAIQNALVNRIVQD 483

CElegans_Vcl NRLLGLCSDIDRLAAQLADLERRGLGNSPEAHQIRNQLKNALRDLGDFMRRVLTDRVVDD 494

: *. :::* :. *. * *: :* . : :* :: .:.:.: :

Zebrafish_VclB FSDTTTPIKLLAVAATAPSDSPNREEVFKERASNFENHASRLGATAEKAAAVGT-ANKST 657

Zebrafish_VclA FSDTTTPIKLLAVAATAPLEAPNREEVFEERASNFENHASRLGATAEKAAAVGT-ANKST 658

Xenopus_Vcl FSDTTTPIKLLAVAATSPSDTPNRDEVFEERATNFESHSARLGATAEKAAAVGS-ANKAT 657

Human_Vcl FSDTTTPIKLLAVAATAPPDAPNREEVFDERAANFENHSGKLGATAEKAAAVGT-ANKST 657

Mouse_Vcl FSDTTTPIKLLAVAATAPPDAPNREEVFDERAANFENHSGRLGATAEKAAAVGT-ANKST 657

Chicken_Vcl FSDTTTPIKLLAVAATAPSDTPNREEVFEERAANFENHAARLGATAEKAAAVGT-ANKTT 657

Drosophila_Vcl FMDVSTPLKQFTEAVLQPEGTPGREQNFNQKSNNLQAFSDRASKTSRMVAAGGACGNKKI 543

CElegans_Vcl FADITTPLKQFVEAVHADPYDPNREQNFVDKSQRLTDHSQSMTTTARLVASCGPSKSKKT 554

* * :**:* :. *. * *:: * ::: .: .: *:. .*: * .*

Zebrafish_VclB VEGIQAAVKSARDLTPQVVSAARILLKNPGNQAAFEHFETMKNQWIDNVEKMTGLVDEAI 717

Zebrafish_VclA VEGIQAAVKSARDLTPQVTSAARILLKNPGNQAAYEHFDTMKNQWIDNIEKMTSLVDEAI 718

Xenopus_Vcl VEGIQAAVKSARELTPQVVSAARILLRNPGNQAAYEHFETMKNQWIDNVEKMTGLVDEAI 717

Human_Vcl VEGIQASVKTARELTPQVVSAARILLRNPGNQAAYEHFETMKNQWIDNVEKMTGLVDEAI 717

Mouse_Vcl VEGIQASVKTARELTPQVISAARILLRNPGNQAAYEHFETMKNQWIDNVEKMTGLVDEAI 717

Chicken_Vcl VEGIQATVKSARELTPQVVSAARILLRNPGNQAAYEHFETMKNQWIDNVEKMTGLVDEAI 717

Drosophila_Vcl AEILLSSAAQVDSLTPQLISAGRIRMNYPGSKAADEHLQNLKQQYADTVLRMRTLCDQAT 603

CElegans_Vcl VEAILDTAEKVEQLTPQLVNAGRVRLHNPGSE---QHFENIHKQYADALHRLRSHVDDAI 611

.* : :. . .****: .*.*: :. **.: :*::.:::*: * : :: *:*

Zebrafish_VclB DTRSLLAASEDAIKKDLDKCQVAMANHQPQMLVAGATSIARRANRILLVAKREIENSEDP 777

Zebrafish_VclA DTKSLLDASEEAIKKDIDKCRVAMANVQPQMLVAGATSIARRANRVLLVAKREVENSEDP 778

Xenopus_Vcl DTRSLLDASEEAIKKDIDKCKVAMANMQPQMLVAGATSIARRANRILLVAKREMENSEDP 777

Human_Vcl DTKSLLDASEEAIKKDLDKCKVAMANIQPQMLVAGATSIARRANRILLVAKREVENSEDP 777

Mouse_Vcl DTKSLLDASEEAIKKDLDKCKVAMANIQPQMLVAGATSIARRANRILLVAKREVENSEDP 777

Chicken_Vcl DTKSLLDASEEAIKKDLDKCKVAMANMQPQMLVAGATSIARRANRILLVAKREVENSEDP 777

Drosophila_Vcl DPADFIKTSEEHMQVYAKLCEDAIHARQPQKMVDNTSNIARLINRVLLVAKQEADNSEDP 663

CElegans_Vcl DTGEFVRASETAMRRYTNHCEGAINGADAHGLVNNSSQIARLGNRVLMTAQNEADNSEEP 671

* .:: :** :: . *. *: : : :* ::.*** **:*:.*:.* :***:*

Zebrafish_VclB KFRETVKAASDELSRTISPMVMDAKAVAANIKDQGLQRGFLDSGFKILGAVANVRDAFQP 837

Zebrafish_VclA KFRELVKAASDELGRTISPMVMAAKGVAGNIQDPGLQKGFLDSGYRILAAVGKVREAFQP 838

Xenopus_Vcl KFRDAVKNASDELSKTISPMVMEAKAVAGNISNPALQKGFLDSGYRILGAVAKVREAFQP 837

Human_Vcl KFREAVKAASDELSKTISPMVMDAKAVAGNISDPGLQKSFLDSGYRILGAVAKVREAFQP 837

Mouse_Vcl KFREAVKAASDELSKTISPMVMDAKAVAGNISDPGLQKSFLDSGYRILGAVAKVREAFQP 837

Chicken_Vcl KFREAVKAASDELSKTISPMVMDAKAVAGNISDPGLQKSFLDSGYRILGAVAKVREAFQP 837

Drosophila_Vcl VFTERLNAAANRLERSLPAMVGDAKLVATNIADPAAAAAWKNSFQRLLGDVREVRDAIAP 723

CElegans_Vcl SFVSRVRNAADQLHNAIPPMVNNAKQIAQNPHDQYAAQNWRGTNDHLLNSVRAVGDAITG 731

* . :. *::.* .:: ** ** :* * : : : ::* * * :*:

Zebrafish_VclB QEP-----------------E--------------FPPPPPDLESL---QISDTAAPPKP 863

Zebrafish_VclA QEL-----------------D--------------FPPPPPDLDQL---HVNDDQAPPKP 864

Xenopus_Vcl PEP-----------------E--------------FPP-PPDLDQL---RLSDEAAPPKP 862

Human_Vcl QEP-----------------D--------------FPPPPPDLEQL---RLTDELAPPKP 863

Mouse_Vcl QEP-----------------D--------------FPPPPPDLEQL---RLTDELAPPKP 863

Chicken_Vcl QEP-----------------D--------------FPPPPPDLEHL---HLTDELAPPKP 863

Drosophila_Vcl P--------------------QPPPLPTS------LPPPIPELSALHLSNQNAERAPPRP 757

CElegans_Vcl VPMSNGRHSSYQESISRASPYNPPPPSSQVIRSVNASPPTAPIIHNKMIIREDIPAPPRP 791

* : ***:*

Zebrafish_VclB PLPEGEVPPPRPPPPEEKDEEFPE------QQAGEMVSEPMMVAARQLHDEARKWSSKGN 917

Zebrafish_VclA PLPEGEVPPPRPPPPEEKDEEFPE------QKAGEMVSEPMMVAARSLHDEARKWSSKGN 918

Xenopus_Vcl PLPEGEVPPPRPPPPEEKDEEFPE------QKVGEVVNQPMMVAARQLHDEARKWSSKGN 916

Human_Vcl PLPEGEVPPPRPPPPEEKDEEFPE------QKAGEVINQPMMMAARQLHDEARKWSSKGN 917

Mouse_Vcl PLPEGEVPPPRPPPPEEKDEEFPE------QKAGEVINQPMMMAARQLHDEARKWSSKGN 917

Chicken_Vcl PLPEGEVPPPRPPPPEEKDEEFPE------QKAGEAINQPMMMAARQLHDEARKWSSKGN 917

Drosophila_Vcl PLPREGLAPVRPPPPETDDEDEG---VFRT---MPHANQPILIAARGLHQEVRQWSSKDN 811

CElegans_Vcl PPPVELSPPPRPPPPPEYDEEEETRAFWERYPLPQASHQPMLAAAHNLHNELKQWSSQEN 851

* * * ***** **: :*:: **: **:* ::***: *

Zebrafish_VclB DIIGAAKRMALLMAEMSRLVRG-SGGNKRALIQCAKDIAKASDEVTRLAKEVAKQCTDKR 976

Zebrafish_VclA DIIGAAKRMALLMAEMSRLVRG-GSGNKRALIQCAKDIAKASDEVTRLAKEVAKQCTDKR 977

Xenopus_Vcl DIIAAAKRMALLMAEMSRLVRG-GSGNKRALIQCAKDIAKASDEVTKLAKEVAKQCTDKR 975

Human_Vcl DIIAAAKRMALLMAEMSRLVRG-GSGTKRALIQCAKDIAKASDEVTRLAKEVAKQCTDKR 976

Mouse_Vcl DIIAAAKRMALLMAEMSRLVRG-GSGTKRALIQCAKDIAKASDEVTRLAKEVAKQCTDKR 976

Chicken_Vcl DIIAAAKRMALLMAEMSRLVRG-GSGNKRALIQCAKDIAKASDEVTRLAKEVAKQCTDKR 976

Drosophila_Vcl EIIAAAKRMAILMARLSELVLSDSRGSKRELIATAKKIAEASEDVTRLAKELARQCTDRR 871

CElegans_Vcl DIVAAAKRMAILMARLSQLVRG-EGGTKKDLINCSKAIADSSEEVTRLAVQLARLCTDIK 910

:*:.******:***.:*.** . *.*: ** :* **.:*::**:** ::*: *** :

Zebrafish_VclB IRTNLLQVCERIPTISTQLKILSTVKATMLGRTNI---------SEEESEQATEMLVHNA 1027

Zebrafish_VclA IRTNLLQVCERIPTISTQLKILSTVKATMLGRTNI---------SEEESEQATEMLVHNA 1028

Xenopus_Vcl IRTNLLQVCERIPTISTQLKILSTVKATMLGRTNI---------SDEESEQATEMLVHNA 1026

Human_Vcl IRTNLLQVCERIPTISTQLKILSTVKATMLGRTNI---------SDEESEQATEMLVHNA 1027

Mouse_Vcl IRTNLLQVCERIPTISTQLKILSTVKATMLGRTNI---------SDEESEQATEMLVHNA 1027

Chicken_Vcl IRTNLLQVCERIPTISTQLKILSTVKATMLGRTNI---------SDEESEQATEMLVHNA 1027

Drosophila_Vcl IRTNLLQVCERIPTIGTQLKILSTVKATMLGA-----------QGSDEDREATEMLVGNA 920

CElegans_Vcl MRTALLQVSERIPTIATQLKVLSTVKATMLGSANVIGPYGQPVEGSEEDDEAMQQLVHNA 970

:** ****.******.****:********** ..:*. :* : ** **

Zebrafish_VclB QNLMQSVKETVREAEAASIKIRTD-AGFTLHWVRKTPWYQ- 1066

Zebrafish_VclA QNLMQSVKETVREAEAASIKIRTD-AGFTLRWVRKTPWYQ- 1067

Xenopus_Vcl QNLMQSVKETVREAEAASIKIRTD-AGCTLRWARKTPWYQ- 1065

Human_Vcl QNLMQSVKETVREAEAASIKIRTD-AGFTLRWVRKTPWYQ- 1066

Mouse_Vcl QNLMQSVKETVREAEAASIKIRTD-AGFTLRWVRKTPWYQ- 1066

Chicken_Vcl QNLMQSVKETVREAEAASIKIRTD-AGFTLRWVRKTPWYQ- 1066

Drosophila_Vcl QNLMQSVKETVRAAEGASIKIRSDQTSNRLQWVRRQPWYQY 961

CElegans_Vcl QNLMQSVKDVVRAAEAASIKIRTN-SGLRLRWLRKPMWSNF 1010

********:.** **.******:: :. *:* *: * :

**S1 Text. Multiple sequence alignment of vinculin proteins**

Multiple sequence alignment of vinculin protein sequences from common model organisms using Clustal Omega. Stars represent complete conservation of the amino acid residue across all sequences. Dots and colons represent almost complete conservation, with differences in one or two residues respectively. The Used NCBI reference sequences: *Drosophila* (NP_476820.1), *C. elegans* (NP_501104.2), Human (NP_003364.1), Mouse (NP_033528.3), Chicken (NP_990772.1), *Xenopus* (NP_001090722.1). Zebrafish vinculin sequences were determined from our own cDNA clones.
